# Immune-Phenotyping and Transcriptomic Profiling of Peripheral Blood Mononuclear Cells From Patients With Breast Cancer: Identification of a 3 Gene Signature Which Predicts Relapse of Triple Negative Breast Cancer

**DOI:** 10.3389/fimmu.2018.02028

**Published:** 2018-09-11

**Authors:** Gemma A. Foulds, Jayakumar Vadakekolathu, Tarek M. A. Abdel-Fatah, Divya Nagarajan, Stephen Reeder, Catherine Johnson, Simon Hood, Paul M. Moseley, Stephen Y. T. Chan, A. Graham Pockley, Sergio Rutella, Stephanie E. B. McArdle

**Affiliations:** ^1^John van Geest Cancer Research Centre, School of Science and Technology, Nottingham Trent University, Nottingham, United Kingdom; ^2^Clinical Oncology Department, Nottingham University Hospitals, Nottingham, United Kingdom

**Keywords:** flow cytometry, breast cancer, nanostring, MDSC, treg, monocytes, NK cells, immune gene signature

## Abstract

**Background:** Interactions between the immune system and tumors are highly reciprocal in nature, leading to speculation that tumor recurrence or therapeutic resistance could be influenced or predicted by immune events that manifest locally, but can be detected systemically.

**Methods:** Multi-parameter flow cytometry was used to examine the percentage and phenotype of natural killer (NK) cells, myeloid-derived suppressor cells (MDSCs), monocyte subsets and regulatory T (Treg) cells in the peripheral blood of of 85 patients with breast cancer (50 of whom were assessed before and after one cycle of anthracycline-based chemotherapy), and 23 controls. Transcriptomic profiles of peripheral blood mononuclear cells (PBMCs) in 23 patients were generated using a NanoString gene profiling platform.

**Results:** An increased percentage of immunosuppressive cells such as granulocytic MDSCs, intermediate CD14^++^CD16^+^ monocytes and CD127^neg^CD25^high^FoxP3^+^ Treg cells was observed in patients with breast cancer, especially patients with stage 3 and 4 disease, regardless of ER status. Following neoadjuvant chemotherapy, B cell numbers decreased significantly, whereas monocyte numbers increased. Although chemotherapy had no effect on the percentage of Treg, MDSC and NK cells, the expression of inhibitory receptors CD85j, LIAR and NKG2A and activating receptors NKp30 and NKp44 on NK cells increased, concomitant with a decreased expression of NKp46 and DNAM-1 activating receptors. Transcriptomic profiling revealed a distinct group of 3 patients in the triple negative breast cancer (TNBC) cohort who expressed high levels of mRNA encoding genes predominantly involved in inflammation. The analysis of a large transcriptomic dataset derived from the tumors of patients with TNBC revealed that the expression of *CD163, CXCR4, THBS1* predicted relapse-free survival.

**Conclusions:** The peripheral blood immunome of patients with breast cancer is influenced by the presence and stage of cancer, but not by molecular subtypes. Furthermore, immune profiling coupled with transcriptomic analyses of peripheral blood cells may identify patients with TNBC that are at risk of relapse after chemotherapy.

## Introduction

Breast cancer accounts for approximately 15% of all cancers and remains the most common and leading cause of cancer deaths in European women (1). Despite earlier detection and significant advances in adjuvant therapy (AT) improving patient outcomes, disease recurrence continues to occur in up to 2% of patients, with approximately 11,000 women succumbing to the disease each year in the UK alone. It is therefore essential that we better understand the drivers of disease, disease recurrence and mechanisms underlying therapeutic resistance.

Triple negative breast cancer (TNBC) encompasses a heterogeneous group of cancers, the treatment of which remains a challenge due to the absence of targetable molecules such as estrogen receptor (ER), progesterone receptor (PR), or epidermal growth factor receptor 2 (HER2). However, the observation that patients with TNBC showing more than 50% lymphocytic infiltration in their tumor core, or within the stroma, exhibit a better prognosis indicates the strong influence of the immune system on disease outcomes (2). Whether the favorable clinical outcome observed in this patient subgroup is related to an inherent ability of the patient to respond to treatment, the quality of the anti-tumor immune response, the functional orientation of the cell infiltrate generated, and/or the immunoregulatory nature of the tumor microenvironment, remains a matter of debate.

Breast cancer does not develop via a single common pathogenic pathway, but encompasses a group of neoplastic events and molecular alterations in breast epithelial cells. In addition to driving the disease, these events can modulate immune responses and the immune contexture of the tumor microenvironment. Consequently, the predictive and prognostic value of tumor infiltrating lymphocytes (TILs) depends on the breast cancer subtype (3). Specific gene expression profiling has enabled the classification of breast cancers into distinct “intrinsic” subtypes (4), with basal-like breast cancers being subdivided into six molecular subtypes, one of which is termed “immunomodulatory” TNBC (5). Furthermore, high intra-tumoral levels of immune-related genes, including those associated with type I interferon responses, and the presence of CD8^+^ cytotoxic T lymphocytes, correlate with improved disease outcome in patients with ERBB2+ breast cancer and TNBC (6, 7).

Tumors can establish and promote an immunoregulatory environment / context which can manifest as detectable changes in the proportion and phenotype of effector and regulatory cell populations in the tumor and periphery. A variety of immune cell types play a critical role in contributing to cancer-induced immune suppression and sustaining cancer progression (8). For example, there is an increased percentage of immunoregulatory CD4^+^CD25^high^FoxP3^+^ T (Treg) cells in the blood and tumors of patients with invasive breast cancer (9). However, it is likely that the number of Treg cells in the blood underestimates the number of these cells present within the tumor microenvironment. Treg cells might also express different activation and chemokine receptors than their peripheral blood counterparts (10).

Given the reciprocal relationship between tumors and the immune system, we have determined the percentage and phenotype of NK cells and immunosuppressive cell types such as non-conventional and intermediate monocytes, Treg cells and myeloid-derived suppressor cells (MDSCs) in the peripheral blood of patients with newly-diagnosed breast cancer and individuals with no-known disease (controls) using multi-parameter flow cytometry, to test the hypothesis that their presence correlates with the intrinsic subtype or with patient prognosis. Since a proportion of patients with breast cancer in our cohort received neoadjuvant chemotherapy prior to surgery, the effect of a single cycle of anthracycline therapy on immune cell phenotypes was also investigated. We also profiled the immune gene transcriptome of peripheral blood mononuclear cells (PBMCs) from a randomly selected subgroup of 24 patients with luminal-A breast cancer and TNBC using the NanoString nCounter™ FLEX amplification-free gene expression profiling platform.

Patients, especially those in stage 3 and 4, exhibited a more immunosuppressive phenotype compared to control volunteers, with increased percentage of granulocytic MDSCs, intermediate CD14^++^CD16^+^ monocytes and CD127^neg^CD25^high^FoxP3^+^ Treg cells, regardless of ER status. Although neoadjuvant chemotherapy had no effect on the percentage of Treg, MDSC and NK cells, the absolute number of B cells decreased and the NK cell receptor profile was altered, with an increase in expression of the inhibitory receptors CD85j, LIAR, and NKG2A and the activating receptors NKp30 and NKp44 as assessed by the MFI of the these marker, and coincided with a decrease in the expression (MFI) of the activating receptors NKp46 and DNAM-1. Transcriptomic profiling revealed that a distinct group of 3 patients with TNBC expressed high levels of mRNA encoding genes predominantly involved in inflammation. The expression of three of these genes (*CD163, CXCR4, THBS1*) was found to predict relapse-free survival in a large, publically-available transcriptomic dataset generated from the tumors of patients with TNBC, but not other breast cancer subtypes. It is therefore possible that TNBC patients with increased risk of relapse as identified with this new gene-signature would benefit from an immunotherapeutic intervention prior to chemotherapy.

## Materials and methods

### Patient cohort

Two cohorts of individuals were retrospectively studied. One study cohort was defined as “no-known disease” (ND) controls (23 women). The other group comprised patients with breast cancer (*n* = 90), the demographics of which are provided in Table 1 and Supplementary Table 1. Informed consent was obtained from all patients and controls, as per local clinical practice. The study was approved by the Institutional Review Board or Independent Ethics Committee and the Hospital Research and Innovations Department at the participating sites. Those patients who received chemotherapy had a sample of blood taken before and 3 weeks after receiving their first chemotherapeutic cycle which consisted of 500 mg/m^2^ 5-fluorouracil, 75–100 mg/m^2^ epirubicin and 500 mg/m^2^ cyclophosphamide.

**Table 1:** Demographics of patients and healthy (no-disease) controls.

		**Cancer patients (Pre-neoadjuvant treatment)**	**Cancer with follow up data (Post first round of neoadjuvant treatment)**	**Healthy volunteers**
*n* =		90	50	23
Mean age (years)		55 (28–89)	52.06 (35–73)	49.387 (35–65)
Tumor grade	1	11		
	2	43		
	3	36		
Tumor stage	I	24		
	II	35		
	III	10		
	IV	21		
ER status	Negative	21		
	Positive	69		
PR status	Negative	38		
	Positive	52		
HER2 status	Negative	75		
	Positive	15		
Phenotype	Luminal A	57		
	Luminal B	12		
	TNBC	18		
	HER2+	3		

### Blood collection and processing

Peripheral blood (5–20 ml) was collected by venipuncture into EDTA-coated Vacutainer™ tubes and processed within 2 h. An aliquot of whole blood was used to measure absolute cell counts using BD Trucount™ beads (BD Biosciences; Mountain View, CA), as per the manufacturer's protocol. Briefly, 100 μL of blood was mixed directly in the BD Trucount™ bead tube and T cell populations, B cells and NK cells were identified using fluorescently-conjugated monoclonal antibodies to CD3, CD8, CD19, CD56, CD14, CD4 and CD45 (details in Supplementary Table 2). Samples were incubated for 15 min at room temperature, protected from the light, and erythrocytes were lysed using BD Pharm Lyse™ (BD Biosciences, 15-min incubation at room temperature). Cells were analyzed by flow cytometry within 1 h.

From the remaining blood, peripheral blood mononuclear cells (PBMCs) were isolated using Ficoll (Greiner Bio-One Ltd, Stonehouse, UK), as per the manufacturer's instructions. Harvested cells were washed twice in phosphate buffered saline (PBS) and viable cells counted using a haemocytometer or ChemoMetec NC250™ cell counter. Freshly isolated PBMCs (2 × 10^6^) were stained using fluorescently-labeled monoclonal antibodies (mAbs) to CD33, HLA-DR, Lineage Cocktail (CD3, CD14, CD19, CD20, CD56), CD11b, CD15, Arginase-1 and CD14 (details in Supplementary Table 2), and the remaining cells were frozen in liquid nitrogen at a density of 10^7^ cells/mL in 90% v/v fetal bovine serum (FBS, Fisher Scientific) and 10% v/v DMSO (Sigma-Aldrich UK, now Merck) for later analysis.

### Immunophenotypic staining of peripheral blood mononuclear cells (PBMCs)

PBMCs were rapidly defrosted and suspended in 10 mL of warmed CTL™ thaw solution (45 mL RPMI 1640 medium, 5 mL CTL™ wash supplement [CTL-Europe GmbH, Bonn, Germany] and 10 μL benzonase), centrifuged (400 g, 5 min) and re-suspended in 2 mL of CTL™ wash solution (45 mL RPMI 1640 medium and 5 mL CTL^TM^ wash supplement). After counting, cells were rested in the incubator for 1–2 h at 37°C.

Frozen and thawed PBMCs were used for further analysis of monocytic MDSCs, Treg cells and NK cells. Any analysis requiring intracellular staining used 2 × 10^6^ cells, whereas the analysis of surface markers only used 1 × 10^6^ cells.

Cells were washed in PBS and incubated with Human FcR Blocking Reagent (MACS™; Miltenyi Biotec) prior to the addition of antibodies directed against cell surface antigens. Analysis of the populations was undertaken using appropriate panels of the following conjugated mAbs: HLA-DR, CD33, CD15, CD11b, CD33, CD14, HLA-DR, CD16, CD3, CD8 (SK1), CD19, DNAM1, NKp46, NKp44, NKp30, 2B4, ICOS, CD14, Lineage Cocktail (CD3, CD14, CD19, CD20, CD56), CD45, NKG2A, CD3, CD25, CD4, LAIR, CD8, CD85j, CD39, NKG2D, and CD127 (details in Supplementary Table 2). LIVE/DEAD™ Fixable Violet Dead stain (Invitrogen) was used to exclude non-viable cells from the analyses.

Analysing the presence of MDSCs and Treg cells required cells to be subsequently fixed and permeabilized (Fixation/Permeabilization kit, eBioscience) before staining with arginase-1 (MDSC staining panel only, R&D Systems) or FoxP3 mAbs (Treg staining panel only, eBioscience) (antibody details in Supplementary Table 2).

Once staining was complete, cells were washed in PBS, re-suspended in Coulter Isoton™ diluent and then analyzed on a 10-color/3-laser Beckman Coulter Gallios™ flow cytometer using Kaluza™ v1.3 acquisition and analysis software (Beckman Coulter).

### Gene expression profiling

A NanoString nCounter™ FLEX platform (NanoString Technologies Inc.) was used to determine immune transcriptomic profiles in PBMCs (11). The nCounter™ analysis system is a robust and highly reproducible method for detecting the expression of up to 800 genes in a single reaction with high sensitivity and linearity across a broad range of expression levels. The platform is based on digital detection and direct molecular barcoding of individual target molecules using a unique probe pair carrying 35- to 50-base target-specific sequences. This technology allows for direct multiplexed measurements of gene expression from a low amount of mRNA (25–300 ng) without the need for amplification by PCR.

For the analysis, total RNA was extracted from frozen PBMCs (5 × 10^6^–10^7^ cells) using a Qiagen RNeasy kit according to the manufacturer's protocol. Quality control of the isolated RNA was performed using an Agilent bioanalyser and NanoDrop 8000 (Thermo Scientific). An OD_260/280_ ratio between 1.8 and 2.2 and a RIN value above 9.0 was considered for further processing.

The RNA Pan-Cancer Immune Profiling Panel, which includes 770 genes (109 cell surface markers for 24 immune cell types, 30 cancer testis antigens, >500 immune response genes, and 40 reference genes) was used. Briefly, 150 ng of RNA was hybridized for 16 h using capture and reporter probes. The samples were then immobilized into a cartridge using the nCounter™ prep-station. Digital images were processed within the nCounter™ Digital Analyser instrument, with 555 fields of view (fov) being collected for each sample. The reporter probe counts, i.e., the number of times the color-coded barcode for that gene is detected, were tabulated in a comma separated value (CSV) format for analysis using the nSolver™ package (version 3.0) and Advanced Analysis module (version 1.0.36). The QC, normalization, differential expression and pathway analysis were performed using the nSolver advance analysis module according to the guidance given by manufacturers (12).

Principle component analysis (PCA) was used for dimensionality reduction and for assessing sample grouping. Genes with a false discovery rate below 0.05 were considered as being significantly differentially expressed.

### Statistical analysis

Statistical analyses were performed using GraphPad Prism 6.0 software. Non-parametric unpaired *t*-test and Mann-Whitney *U*-test were used to assess the significance of differences between healthy controls and patients with breast cancer. A paired *t*-test was used to determine the significance of differences between the same patients before and after chemotherapy. A non-parametric One-Way ANOVA and Kruskal-Wallis test were used when assessing more than two groups of data. Multiple comparisons were also made between the medians of each data set. For all statistical comparisons, a *p*-value < 0.05 was considered to represent statistically significant differences.

## Results

The underlying hypothesis of the study is that phenotypic profiling and immune transcriptomic analyses of peripheral blood cells reflect disease status in patients with breast cancer and have the potential to inform clinical decisions and help predict therapeutic response. We therefore investigated (1) whether the peripheral blood immunome of breast cancer patients differs from that of healthy controls; (2) whether chemotherapy affects this immune phenotype; and (3) whether a defined peripheral blood immune phenotypic profile relates to a specific molecular breast cancer subgroup.

### The peripheral blood immunome of patients with breast cancer displays a more suppressive phenotype than that of age-matched controls

The frequency and phenotype of T cells, B cells, monocyte subsets and NK cells in the peripheral blood of 67 patients with breast cancer and in 23 age/sex-matched healthy controls was assessed. The clinical and biological characteristics of the patients are shown in Table 1 (More details in Supplementary Table 1). The majority of patients had luminal-A breast cancer (46 of 67) and stage I-II disease (45 of 67).

**Figure 1:**
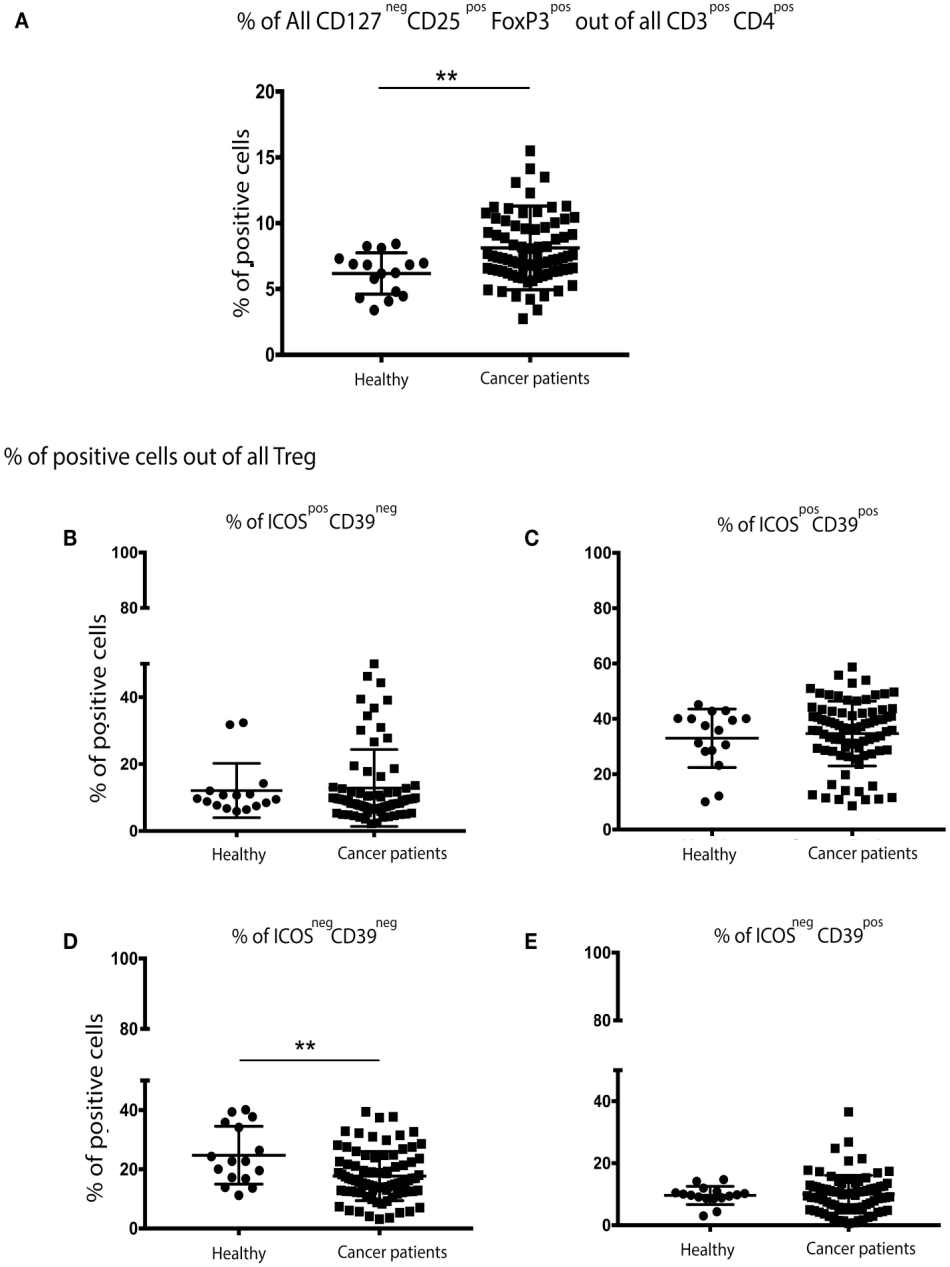
There are significantly more Treg cells in the peripheral blood of patients with breast cancer and these exhibit an overall more activated/suppressive phenotype: PBMCs from patients with breast cancer and individuals with no-known disease were rapidly defrosted, allowed to rest for 2 h at 37°C, washed, and incubated with an Fc blocking reagent before being stained with a cocktail of mAbs reactive with cell surface antigens. Intracellular staining was undertaken following treatment with Fix/Perm and incubation with a FoxP3 mAb. Data were acquired on a Beckman Coulter Gallios™ flow cytometer and analyzed using Beckman Coulter Kaluza™ software. A two-tailed Mann Whitney test was performed to determine any significant differences in the measured parameters between healthy individuals and patients with breast cancer. A Wilcoxon matched-pairs two tailed test was used to assess the significance between Pre/Post-chemotherapy. A Kruskal-Wallis test was used to assess the significance of any differences in the measured parameters between the different disease stages, and individuals with no-known disease (^**^P < 0.005). There was a significantly higher number of CD3^+^CD4^+^CD127^neg^CD25^+^FoxP3^+^ (regulatory T (Treg) cells) in the PBMCs of patients with breast cancer **(A)**. No differences were found between healthy controls and cancer patients for CD39^neg^/ICOS^pos^ **(B)** or CD39^pos^/ICOS^pos^ **(C)** or CD39^pos^/ICOS^neg^ **(E)**, however there were significantly fewer CD39^neg^/ICOS^neg^ Treg cells in the peripheral blood of patients with breast cancer **(D)**.

The percentage of CD3^+^CD4^+^CD127^neg^CD25^+^FoxP3^+^ Treg cells were significantly higher in patients with breast cancer (Figure 1A) than in their no-known disease, age-matched counterparts. We then focused on a more in-depth characterization of Treg cell subsets. CD39 expression has previously been shown to be restricted to a subset of FoxP3^+^ regulatory effector/memory-like T (TREM) cells, whereas ICOS is a CD28-like co-stimulatory molecule which is expressed by recently-activated T cells (13). We found that the proportion of Treg cells expressing CD39 and ICOS, either alone or in combination was the same in patients with breast cancer and controls (Figures 1B,C,E). However, the proportion of Treg cells lacking both CD39 and ICOS expression was higher in patients compared to controls (Figure 1D).

**Figure 2:**
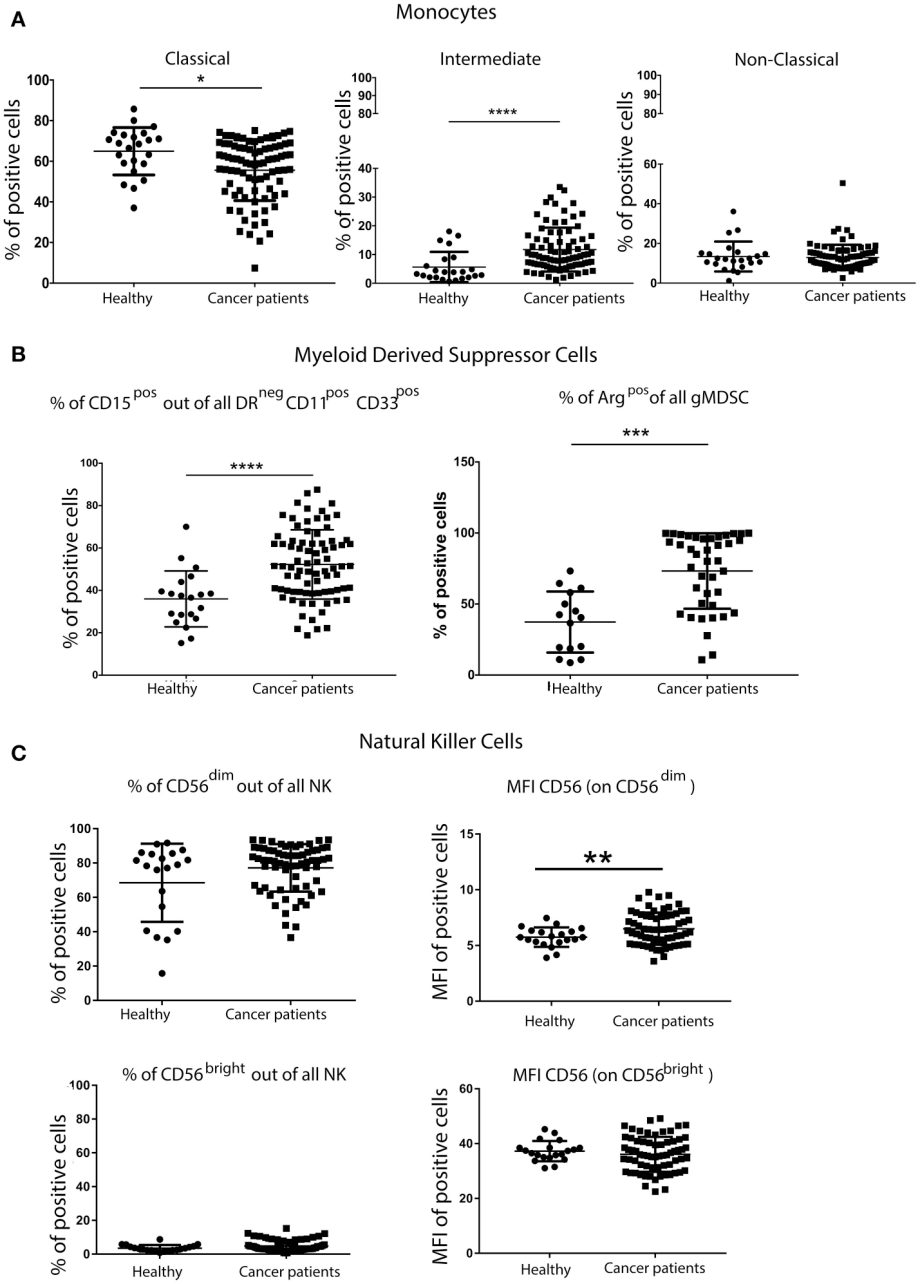
Greater percentage of inflammatory monocytes and granulocytic MDSCs (gMDSCs) in the peripheral blood of patients with breast cancer than in age-matched volunteers. PBMCs from patients with breast cancer and individuals with no-known disease were rapidly defrosted, allowed to rest for 2 h at 37°C, washed and then incubated with an Fc blocking reagent before being stained with a cocktail of mAbs reactive with cell surface antigens. Freshly isolated PBMCs from a proportion of patients were then stained for the expression of intracellular Arginase. Data were acquired using a Beckman Coulter Gallios™ flow cytometer and analyzed using Beckman Coulter Kaluza™ software. A two-tailed Mann Whitney test was performed to assess differences between patients with cancer and their corresponding controls, and a Wilcoxon matched-pairs two tailed test was used to assess the influence of chemotherapy. A Kruskal-Wallis test was used to assess the significance of any differences in the measured parameters between the different disease stages, and individuals with no-known disease (^*^P < 0.05; ^**^P < 0.005; ^***^P < 0.0005). A significantly higher percentage of pro-inflammatory monocytes within the CD14^+^ population was present in the PBMCs from patients with breast cancer **(A)**. Although no difference was found in the proportion of CD11b^+^CD33^+^ within DR^neg^ cells, a significantly higher percentage of DR^neg^CD11b^+^CD33^+^ cells were CD15^+^ in patients with breast cancer **(B)**. A significantly higher percentage of freshly isolated MDSCs from patients with breast cancer were Arginase^+^ cells, and the percentage of Arginase^+^ MDSCs was positively associated with disease stage **(B)**. Similar proportions of CD56^dim^CD16^+^ and CD56^bright^CD16^−^ NK cells were present in patients with breast cancer and healthy controls; however there were significant differences in the intensity of CD56 expression on both NK cell subsets **(C)**.

Although there were no differences in the overall percentage of all monocytes in the blood of patients and controls (data not shown), more detailed analysis of monocyte subsets revealed fewer classical monocytes (CD14^++^CD16^neg^), but significantly more intermediate monocytes (CD14^++/+^CD16^+^) in patients with breast cancer. No differences in the frequency of non-classical monocytes (CD14^+^CD16^++^) in patients and controls were observed (Figure 2A).

PBMCs were also stained with a cocktail of mAbs in order to identify and enumerate MDSCs (antibody details in Supplementary Table 2). Although no difference in the overall frequency of CD11b^+^CD33^+^ within HLA-DR^neg^ cells was found (data not shown), a significantly higher percentage of HLA-DR^neg^CD11b^+^CD33^+^ cells also expressing CD15^+^ was found in the PBMCs of patients with breast cancer compared with controls (Figure 2B). MDSCs in the PBMCs from patients with breast cancer were enriched in potentially immunosuppressive arginase-1^+^ cells. These phenotypic characteristics were consistent with the granulocytic nature of the MDSCs. It has been suggested that granulocytic MDSCs (HLA-DR^neg^CD11b^+^CD33^+^CD15^+^) should only be evaluated in freshly isolated PBMCs (14). However, although some granulocytic MDSCs were lost during freezing/defrosting of PBMC preparations (Supplementary Figure 1), the significantly increased percentage of gMDSCs in the blood of patients with breast cancer compared to healthy controls was maintained (Supplementary Figure 1).

The percentage of NK cells and the proportion of the primary NK cell subsets (CD56^dim^CD16^+^ and CD56^bright^CD16^−^) in patients with breast cancer and controls were not significantly different (Figure 2C). Further analysis of these cell subsets revealed significant differences in the mean fluorescence intensity (MFI) of CD56 expression, with the intensity of CD56 expression being significantly higher on the CD56^dim^CD16^+^ subset (Figure 2C). The intensity of CD16 expression was the same for both NK cell subsets. As NK cells express a variety of inhibitory and activating receptors which are involved in target recognition and killing, the expression levels of nine NK cell receptors were determined in patients with breast cancer. The expression of activating receptors NKp30, NKp46 and 2B4, as detected by the increase in the MFI, was significantly decreased. Although the proportion of NK cells expressing the inhibitory receptor NKG2A was decreased, the intensity of NKG2A expression on cells was increased (Supplementary Figure 2). It is possible that the activity rather the proportion of NK cells is affected by the presence of tumor, indeed, Konjević et.al. found that the activity of NK cells from patients with breast cancer was significantly decreased and correlated with the spontaneous release of LDH by the peripheral blood lymphocytes (PBL) of patients with breast cancer in a stage-dependant manner (15).

### Chemotherapy significantly decreases the absolute number of B cells

The quantification of helper T cells (CD3^+^CD4^+^), cytotoxic T cells (CD3^+^CD8^+^), B cells (CD19^+^CD3^neg^), monocytes (CD45^+^CD14^+^) and NK cells (CD16^+^/CD56^+^CD3^neg^) revealed no significant differences between patients with breast cancer and healthy controls (data not shown). Moreover, the overall percentage of Treg cells, MDSCs and NK cells was not significantly altered following chemotherapy (nor was the relative distribution of the CD56^dim^ and CD56^bright^ subsets). In contrast, a single round of neoadjuvant chemotherapy resulted in significant changes in the MFI of certain NK cells markers. Indeed, the intensity of CD56 expression on CD56^dim^CD16^+^ NK cells was significantly increased, as was the percentage of NK cells expressing the activating receptor NKp44, the intensity of expression (MFI) of activating receptors NKp30 and 2B4 and the expression (MFI) of the inhibitory receptors LAIR and NKG2A. An increase in the percentage of the CD56^bright^ subset of NK cells expressing the inhibitory receptor CD85j, concomitant with a decreased percentage of cells expressing the inhibitory receptor DNAM-1 and the activating receptor NKp46 (although the intensity of expression was also observed to increase on positive cells) (Supplementary Figure 2).

**Figure 3:**
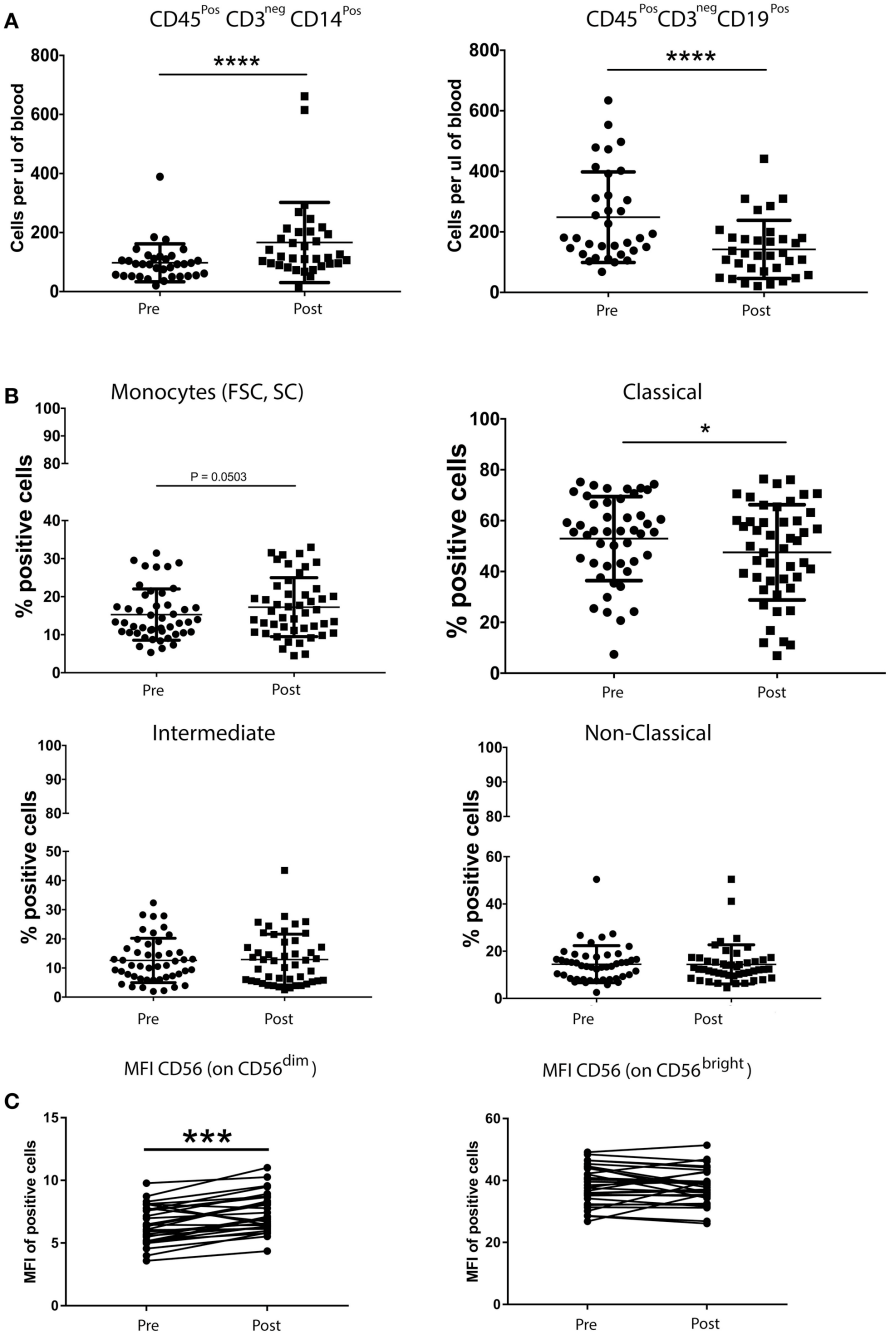
Chemotherapy affects the absolute number of B cells and monocytes **(A)**, but not the percentage of Treg cells, MDSCs and NK cells **(B)**. Peripheral blood (5–20 mL) was collected into EDTA Vacutainers™ and processed within 2 h. An aliquot of whole blood was used to obtain T cell, B cell, monocyte and NK cell counts using BD Trucount™ beads (BD Biosciences) following the manufacturers protocol. For this, 100μL of blood was mixed directly in the BD Trucount™ bead tube with mAbs to CD3, CD8, CD4, CD19, CD56, CD45, and CD14. Tubes were incubated for 15 min at room temperature, protected from light, after which erythrocytes were lysed via a 15-min incubation at room temperature in BD Pharm Lyse™ lysing solution. Cells were analyzed by flow cytometry within 1 h. No significant differences were found in the number of the different immune cells between patients and healthy donors. B cells and monocytes were the only two cell types significantly affected by one course of chemotherapy **(A)**. A paired *t*-test was used to compare the samples. (^*^P < 0.05; ^**^P < 0.005; ^***^P < 0.0005) and **(C)** PBMCs from patients with breast cancer and individuals with no-known disease were rapidly defrosted, allowed to rest for 2 h at 37°C, washed, and incubated with an Fc blocking reagent before being stained with a cocktail of mAbs reactive with cell surface antigens. Intracellular staining was undertaken following treatment with Fix/Perm and incubation with a FoxP3 mAb. Data were acquired using Beckman Coulter Gallios™ flow cytometer and analyzed using Beckman Coulter Kaluza™ software. A two-tailed Mann Whitney test was performed to assess significant differences in the phenotypic profiles in patients with breast cancer and their corresponding controls, and a Wilcoxon matched-pairs two tailed test was used to assess the influence of chemotherapy. A Kruskal-Wallis test was used to assess the significance of any differences in the measured parameters between the different disease stages, and individuals with no-known disease (^*^P < 0.05; ^**^P < 0.005; ^***^P < 0.0005). The percentage of Treg cells was not affected by one round of chemotherapy (data not shown). The abosulte number of monocytes (CD45^+^CD3^neg^CD14^+^) was found to be significantly increased by chemotherapy **(B)** while that of B-cells (CD45^+^CD3^neg^CD19^+^) was significantly decreased, whereas only the MFI of CD56^dim^ NK cells was affected by chemotherapy.

The absolute number of classical monocytes (as assessed by CD45^+^CD3^neg^CD14^+^) was lower after chemotherapy (Figure 3). A statistically significant decrease of B cells and an increase of monocyte counts over baseline values was found after neoadjuvant anthracycline treatment (Figures 3A,B), in line with previous findings by Wijayahadi (16) and Verma (17).

The immune profile of the PBMCs isolated from the blood of patients with breast cancer showed significant differences compared to those isolated from the blood of healthy controls and tended to increase with stage although this was not statistically significant (Supplementary Figure 3).

We hypothesized that the lack of differences between molecular subtypes observed might be caused by the limited number of markers that had been used to characterize Treg cells, and our focused immunophenotyping of MDSCs, monocytes and NK cells and that the assessment of a wider range of immune-related genes would reveal differences between, at least, the most clinically distinct breast cancer subgroups, i.e., luminal-A vs. TNBC.

### Identification of a 3-gene signature which predicts relapse in TNBC treated with chemotherapy

**Figure 4:**
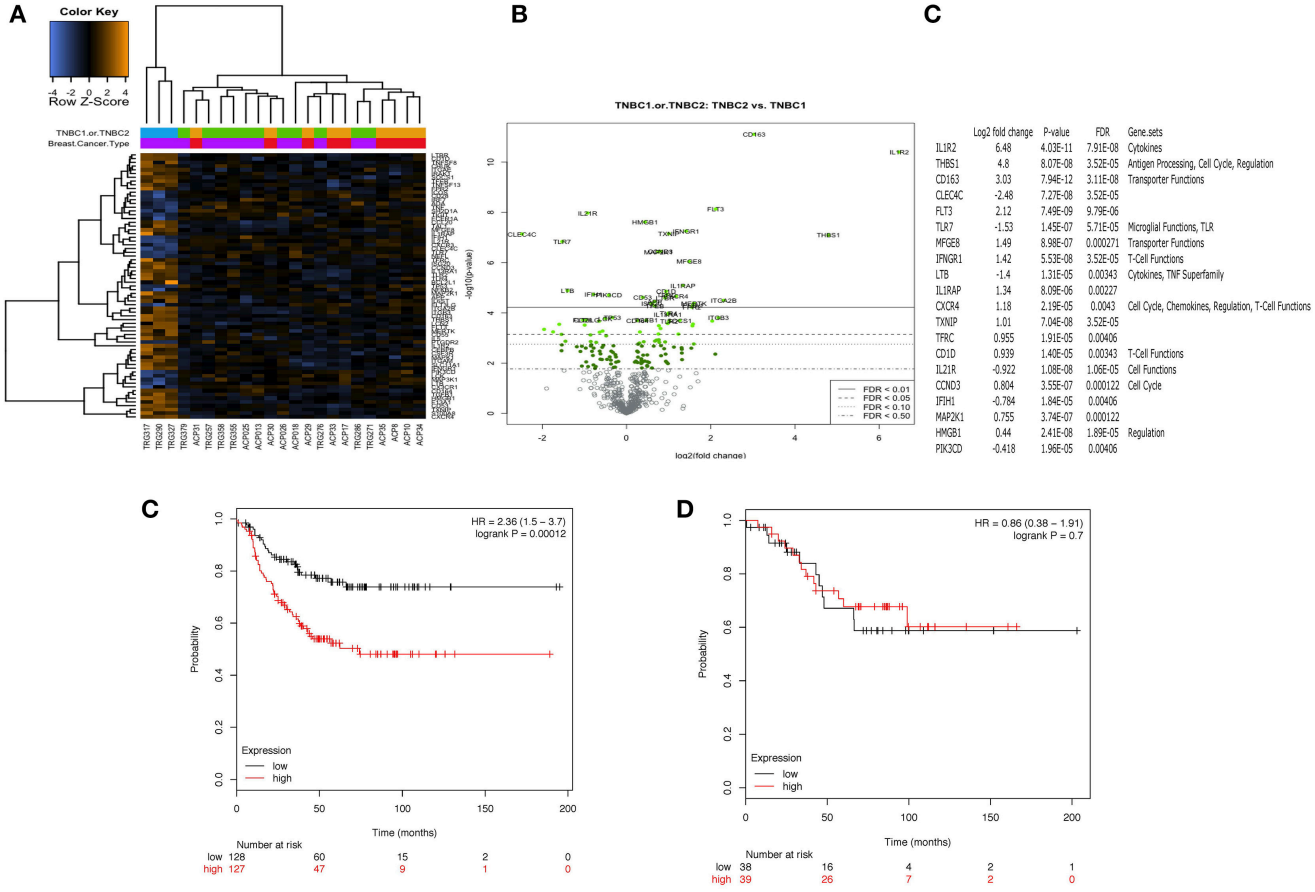
Peripheral blood mononuclear cells (PBMCs) from a distinct subgroup of patients with TNBC differentially expressed immune-related genes. Total RNA was extracted from frozen PBMCs frozen PBMCs from 23 randomly selected patients, 14 with TNBC and 9 with luminal-A breast cancer (5 × 10^6^ – 10^7^ cells) using a Qiagen RNAesasy kit according to the manufacturer's instructions. Gene expression profiling was carried out using a NanoString nCounter™ FLEX platform (NanoString Technologies Inc.). The Pan-Cancer Immune Profiling Panel was used to assess immune function at the mRNA level according to the manufacturer's protocol. Raw data were acquired using the nCounter™ digital analyser with 555 fields of view (fov) being collected for each sample. Differential expression and further analysis was performed using Human PanCancer Immune Profiling Advanced Analysis (version 1.0.36). Principle component analysis (PCA) was used for dimensionality reduction and assessing sample grouping. Genes with a false discovery rate below 0.05 were considered as significant. No differences in the mRNA expression profiles in PBMCs from 9 patients with Luminal A disease and 11 of the patients with TNBC were observed. However, PBMCs from 3 out of the 14 patients with TNBC demonstrated a clearly distinct immune gene expression profile **(A)**. **(B,C)** Show the 20 top most differentially regulated genes between these two groups [PBMCs from 11 patients with TNBC (TNBC1) and the 3 distinct TNBC (TNBC2)]. High expression of *CD163* combined with high expression of *CXCR4* and low expression of *THBS1* was associated with better RFS (*p* = 0.00012). Interestingly, this combination of genes was not associated with any difference in RFS for patients with ER positive breast cancer (*p* = 0.7) **(D)**.

The immune transcriptome from a subgroup of 23 randomly selected patients, 14 with TNBC and 9 with luminal-A breast cancer was profiled using the NanoString nCounter™ platform. Overall, immune gene expression profiles in the PBMCs of patients with TNBC and luminal-A breast cancer were similar (Figure 4A). However, an unsupervised clustering analysis revealed that three of the 14 patients with TNBC exhibited distinct immune gene expression patterns (Figure 4A). A list of the top 20 differentially expressed genes is shown in Figures 4B,C. The gene sets that were up- or down-regulated in these two subgroups of TNBC patients included *CD163* (a scavenger receptor involved in the resolution of inflammation), cytokine receptors such as *IFNGR1* (CD119), *IL21R, IL1R2, FLT3, IL1RAP*, and *TXNIP* (which promotes anti-inflammatory macrophages) and *HMGB1* (which is upregulated under inflammatory conditions) (Figures 4B,C). The top 20 differentially expressed genes were then further analyzed using Metacore software (Supplementary Table 3). Eight of them were found to be functionally related to the inflammation pathway and their ability to predict clinical outcome (when expressed in the tumors of patients) was analyzed *in silico* in 186 TNBC (basal) patients using the Kaplan-Meier-plotter on-line tool (18). The analysis revealed that the combined low-expression of *CD163* and *CXCR4* and the high expression of *THBS1* significantly correlated with having a high risk of recurrence and poor survival rate in TNBC (basal), but not in patients with either ER^+^ or HER^+^ disease (Figure 4E). However, it should be note that these results were obtained using unfractionated PBMCs which may have masked some immune signature.

## Discussion

Herein, we report on the changes in the absolute number of monocytes and B cells 2 weeks after the first cycle of chemotherapy, on the increased percentage of immune suppressive cells such as Treg cells MDSCs, and intermediate monocytes as well as the increased expression of inhibitory receptors on the surface of NK cells (as assessed by the increase in MFI) in the PBMCs of 85 patients with breast cancer (50 of which were evaluated both before and after one cycle of anthracycline-based chemotherapy) and 23 control volunteers. These immune cell types are known to contribute to cancer-induced immune suppression and play a critical role in sustaining cancer progression (8). Moreover, this study has, for the first time, demonstrated that the assessment of 770 immune-related genes in PBMCs from patients with TNBC using the NanoString nCounter™ amplification-free gene expression profiling platform was able to highlight differences in a subset of patients with TNBC which, if further validated in a prospective study, could have extremely high clinical significance for the management and treatment of TNBC. Indeed, monitoring the expression of these 3-genes in patients with TNBC after standard of care could anticipate those at high risk of relapsing who might benefit from a vaccine intended to target any new emerging cancer cells.

Our results reinforce and expand previous studies showing significantly higher percentage of circulating Treg cells and MDSCs in patients with breast cancer, and their association with increased tumor burden (19, 20). Treg cells from the patients evaluated in the study reported herein also displayed a suppressive/activated phenotype compared with those from healthy donors, as was indirectly indicated by the significant decrease in the percentage of Treg cells lacking both ICOS and CD39 expression. This observation suggests that the proportion of ICOS^−^CD39^−^ Treg cells may increase as the disease progresses. Importantly, ICOS^+^FoxP3^+^ Treg cells use IL-10 to suppress the function of dendritic cells (DCS) and TGF-β to suppress T cell function, whereas the ICOS^−^FoxP3^+^ Treg cells primarily employ TGF-β (21, 22). Other cytokines have been shown to be involved in the suppression and progression of cancer, For example, levels of TNFa have been found to increase with the clinical stage in multiple myeloma patients (23). In view of these results, further studies should include additional Treg markers in order to perform a more in depth anlaysis of these cells in the PBMCs of patients with breast cancer, such as those that have been performed by Miyara (24) or Kordasti (25).

In addition to the increased proportion of Treg cells, higher numbers of MDSCs were present in patients with breast cancer, and the percentage of these increased with disease stage, albeit not significantly. This was in agreement with another study which has recently reported on the presence of elevated frequencies of CD14^+^HLA-DR^−/low^ mMDSCs in early breast cancer (26). Importantly, Bailur et al. demonstrated that the ability of women with HER2^+^ breast cancer to mount an immune response against the HER2 antigen was linked with low levels of Lin^−^CD14^+^HLA-DR^−^ MDSCs and Treg cells, and that this was associated with a 100% 5-year survival rate compared to only 38% for those who had no detectable level of Her2-reactive CD8^+^ T cells, but had high frequencies of MDSCs (27). In addition, a study in 17 patients with early stage breast cancer receiving doxorubicin–cyclophosphamide chemotherapy found a correlation between circulating MDSCs, clinical stage, chemotherapy treatment and metastatic tumor burden Gonda et al. (28). A more recent study, (29) found that MDSC levels were increased in pre-operative breast cancer patients, but decreased following removal of their tumor. More importantly, the proportion of MDSCs increased following recurrence. Furthermore, MDSC levels in patients with recurrent breast cancer have been found to decrease following chemotherapy (29). Immunotherapeutic approaches using IL-2 and agonistic CD40 antibodies have been shown to effectively remove both Treg cells and MDSCs. These strategies should be considered prior to, or in combination with, active immunotherapy as a means of depleting immune suppressive cell types, thus favoring durable anti-tumor responses (30).

The expansion of distinct monocyte subsets having a unique phenotype and function has been associated with cardiovascular diseases, inflammation, cancer and the administration of growth factors (31). Moreover, elderly, frail patients with breast cancer who experience unexpected hospitalization after chemotherapy treatment exhibit higher levels of granulocytic cells (CD15^+^), but lower levels of cells with suppressor phenotypes, including CD14^−^CD15^+^CD124^+^ MDSCs and Treg cells, at baseline (32). Herein, we have found a significantly higher number of CD14^++/+^CD16^+^ intermediate monocytes in patients with breast cancer. Interestingly, CD14^+^CD16^+^ monocytes have been proposed to be an early indicator of breast cancer (33).

We also found that chemotherapy significantly decreased the absolute number of B cells, whereas the percentages of suppressive cells such as Treg cells and MDSCs were not affected. This is of particular importance for future immunotherapeutic interventions in patients undergoing chemotherapy, since not only do B cell numbers decrease significantly, as reported by Verma et al. (19), but they remain significantly depleted even 9 months after chemotherapy. Furthermore, their phenotype also remains significantly different from those that were present prior to chemotherapy.

The importance of NK cells in the manifestation of protective anti-tumor immunity is increasingly evident. In mouse models of breast cancer, NOD/SCID/γc^null^ mice (those which lack T, B, and NK cells) implanted with human MDA-MB-231 (triple negative) breast cancer cells exhibited primary tumor formation followed by the formation of metastases about 3 weeks later (34). However, similar experiments conducted in NOD/SCID mice (which possess NK cells) result in smaller primary tumors, but metastatic spread. In a large-scale prospective study in Japan, the cytotoxic activity of NK cells was assessed in 3625 volunteers who were then monitored for cancer development during the following 11 years. Intriguingly, individuals with low NK cell activity at baseline were significantly more susceptible to the development of cancer (35).

The triggering of NK cells by target cells involves a balance between signals from activating and inhibitory receptors. Although previous studies have shown that the expression of some NK cell receptors (NKG2D, NKp30, DNAM-1, CD16, and 2B4) may be low in patients with breast cancer (36), the prognostic or diagnostic value of this information remains unclear. Furthermore, although neoadjuvant therapy reduces NK cell numbers (17), little is known about its effect on the expression of NK cell activating and inhibitory receptors. The effect of chemotherapeutic treatments on the expression of NK cell receptors has not been widely studied in patients with breast cancer. Previous reports have shown that the density of expression of NK cell activating and cytotoxicity receptors, e.g., NKG2D, NKp30, DNAM-1, CD16, and 2B4, may be reduced in patients compared with healthy volunteers (36). In this study, patients displayed significantly reduced expression of the activating receptors NKp30, NKp46, and 2B4, in addition to a significant reduction in expression of the inhibitory receptors NKG2A.

Immune gene expression profiling studies showed that PBMCs from patients with Luminal A disease were not significantly different to those from patients with TNBC. These findings confirmed our flow cytometry data which showed that the more immune-suppressive phenotype observed was disease-related rather than molecular sub-type specific. Interestingly, the concept that the immune phenotype, whether in the periphery or in the tumor microenvironment, correlates more closely with the severity of the disease rather than the breast cancer molecular subtypes has also recently been described by Savas et al. (37) who found that the overall percentage of CD45^+^ TILs did not change according to each breast cancer subtype (TNBC, HER2+, and hormone receptor–positive (luminal)), but was significantly higher in primary than in metastatic tumor samples. In our study, we have found that the transcriptome of PBMCs from 3 of the 14 patients with TNBC which were analyzed showed a significant difference in the expression of genes mostly related to inflammation which, after *in silico* analysis, was reduced to 3 genes (CD163, CXCR4, THBS1). Subsequent analysis using a publicly-available dataset revealed that the coordinated low-expression of *CD163* and *CXCR4* with high expression of *THBS1* in 161 breast cancer tissues identified patients with TNBC (basal) having a high risk of recurrence and poor survival rate. We are now pursuing the validation of this signature in the PBMCs and tissue of the same patients in order to find out whether these genes can be used as a blood-based surrogate of the tumor immune microenvironment and be used to identify patients at risk of recurrence after chemotherapy. However, it should be noted that because the NanoString profiling was perfomed using unfractionated PBMCs this might have masked specific immune signature from immune cell subtype and future studies will need to profile isolated cell sub-populations.

In summary, this study has demonstrated that the peripheral blood immunome in patients with breast cancer exhibits a more immunosuppressive phenotype than that of healthy controls, and that this is more associated with the disease stage than the ER status. Interrogation of the peripheral blood at the cellular and transcriptional level has the potential to provide insight into the presence and stage of disease. Moreover, although the ability of transcripts for CD163/CXCR4 and THBS1 in PBMCs to predict intra-tumoral expression remains to be demonstrated, this novel immune signature could prove extremely important in the monitoring of patients with TNBC. It is indeed conceivable that minimally-invasive analyses of immune gene transcripts in the blood will inform clinical decisions, including the potential use of immunomodulatory agents.

## Availability of data and materials

All relevant data generated or analyzed during this study are included in this published article and its Supplementary Information Files.

## Author contributions

SM and AP conception and design. SC, PM, and TA-F development of methodology, patient consent, blood and clinical data collection. SReeder, CJ, DN, SH, GF, JV, and SM blood separation, flow cytometry staining/acquisition and NanoString experiments. GF, SM, JV, and SR data analysis and interpretation (e.g., analysis of flow cytometry and transcriptomic data). SM, GF, JV, SRutella, AP, TA-F, PM, and SC preparation and revision of the manuscript.

### Conflict of interest statement

The authors declare that the research was conducted in the absence of any commercial or financial relationships that could be construed as a potential conflict of interest.

## Supplementary material

Supplementary Figure 1 Despite the significant loss of gMDSCs after defrosting, significant differences in the percentage of CD15^+^ cells within the DR^neg^CD11b^+^CD33^+^ population between healthy controls and patients is retained. PBMCs from patients with breast cancer and individuals with no-known disease were rapidly defrosted, allowed to rest for 2 h at 37°C, washed and then incubated with an Fc blocking reagent before being stained with a cocktail of mAbs reactive with cell surface antigens. Alternatively, freshly isolated PBMCs from a proportion of patients and healthy individuals were stained using a cocktail of mAbs that define MDSCs. Data were acquired using a Beckman Coulter Gallios™ flow cytometer and analyzed using Beckman Coulter Kaluza™ software. A two-tailed Mann Whitney test was performed to assess differences between patients with cancer and their corresponding controls, and a Wilcoxon matched-pairs two-tailed test was used to assess the influence of chemotherapy. A Kruskal-Wallis test was used to assess the significance of any differences in the measured parameters between the different disease stages, and individuals with no-known disease (^*^*P* < 0.05; ^**^*P* < 0.005; ^***^*P* < 0.0005). The majority of granulocytic MDSCs (DR^neg^CD11b^+^CD33^+^CD15^+^) did not survive the freezing/defrosting procedure (Figure 1A). However, the significantly higher percentage of CD15^+^ cells within the DR^neg^CD11b^+^CD33^+^ population in the PBMCs from patients with breast cancer (compared with PBMCs from age-matched controls) remained apparent in the defrosted PBMCs (Figure 1B), with a significantly higher proportion being apparent in the early stages of disease (Stage1) (Figure 1C).

Supplementary Figure 2 NK cell subsets in the periphery of healthy controls and patients with breast cancer **(A)**, and in patients with breast cancer after one round of chemotherapy **(B)**. Similar proportions of CD56^dim^CD16^+^ and CD56^bright^CD16^−^ NK cells were present in patients with breast cancer and healthy controls **(A)**. Chemotherapy had no effect on the intensity of CD16 expression **(B)**.

Supplementary Figure 3 The percentage of immunosuppressive immune cells increases with tumor stage. PBMCs from patients with breast cancer and individuals with no-known disease were rapidly defrosted, allowed to rest for 2 h at 37°C, washed and then incubated with an Fc blocking reagent before being stained with a cocktail of mAbs reactive with cell surface antigens. Data were acquired using a Beckman Coulter Gallios™ flow cytometer and analyzed using Beckman Coulter Kaluza™ software. A two-tailed Mann–Whitney test was performed to assess differences between patients with cancer and their corresponding controls, and a Wilcoxon matched-pairs two-tailed test was used to assess the influence of chemotherapy. A Kruskal–Wallis test was used to assess the significance of any differences in the measured parameters between the different disease stages, and individuals with no-known disease (^*^*P* < 0.05; ^**^*P* < 0.005; ^***^*P* < 0.0005). Significant differences in the percentage of **(A)** Treg, **(B)** classical (CD14^++^CD16^neg^), or **(C)** intermediate (CD14^++/+^CD16^+^) monocyte subtypes and the percentage of **(D)** MDSCs (DR^neg^CD11b^+^CD33^+^CD15^+^) were primarily found between healthy controls and patients with cancer, rather than between difference molecular subtypes of cancer. However, the majority of patients had luminal disease. Consequently, a subsequent analysis was performed on patients diagnosed with Luminal A only and those with a higher stage disease tended to have less Treg CD39^+^/ICOS^+^, less classical monocytes, and more intermediate monocytes and more gMDSCs.

Supplementary Table 1 Patient demographics and summary of flow cytometric analysis.

Supplementary Table 2 Antibody panels used in this study.

Supplementary Table 3 Identification of an immune gene signature predicting high risk of relapse and poor survival in TNBC. The 20 most differentially regulated genes identified by the nSolver software package (Nanostring Technologies) were assessed by Metacore, the results of which are shown here (Table 3).

## Abbreviations

MDSC(s): myeloid-derived suppressor cell(s)
Treg cell(s): immunoregulatory T cell(s).

## References

[1] Ferlay J Steliarova-Foucher E Lortet-Tieulent J Rosso S Coebergh JW Comber H. Cancer incidence and mortality patterns in Europe: estimates for 40 countries in 2012. Eur J Cancer (2013) 49:1374–403. 10.1016/j.ejca.2012.12.027 23485231

[2] Adams S Gray RJ Demaria S Goldstein L Perez EA Shulman LN. Prognostic value of tumor-infiltrating lymphocytes in triple-negative breast cancers from two phase III randomized adjuvant breast cancer trials: ECOG 2197 and ECOG 1199. J Clin Oncol. (2014) 32:2959–66. 10.1200/JCO.2013.55.0491 25071121 PMC4162494

[3] Ono M Tsuda H Shimizu C Yamamoto S Shibata T Yamamoto H. Tumor-infiltrating lymphocytes are correlated with response to neoadjuvant chemotherapy in triple-negative breast cancer. Breast Cancer Res Treat (2012) 132:793–805. 10.1007/s10549-011-1554-7 21562709

[4] Kroemer G Senovilla L Galluzzi L Andre F Zitvogel L. Natural and therapy-induced immunosurveillance in breast cancer. Nat Med. (2015) 21:1128–38. 10.1038/nm.3944 26444637

[5] Lehmann BD Bauer JA Chen X Sanders ME Chakravarthy AB Shyr Y. Identification of human triple-negative breast cancer subtypes and preclinical models for selection of targeted therapies. J Clin Invest. (2011) 121:2750–67. 10.1172/JCI45014 21633166 PMC3127435

[6] Denkert C Loibl S Noske A Roller M Muller BM Komor M. Tumor-associated lymphocytes as an independent predictor of response to neoadjuvant chemotherapy in breast cancer. J Clin Oncol. (2010) 28:105–13. 10.1200/JCO.2009.23.7370 19917869

[7] Miyashita M Sasano H Tamaki K Chan M Hirakawa H Suzuki A. Tumor-infiltrating CD8+ and FOXP3+ lymphocytes in triple-negative breast cancer: its correlation with pathological complete response to neoadjuvant chemotherapy. Breast Cancer Res Treat (2014) 148:525–34. 10.1007/s10549-014-3197-y 25395319

[8] Mantovani A Allavena P Sica A Balkwill F. Cancer-related inflammation. Nature (2008) 454:436–44. 10.1038/nature07205 18650914

[9] Liyanage UK Moore TT Joo HG Tanaka Y Herrmann V Doherty G Prevalence of regulatory T cells is increased in peripheral blood and tumor microenvironment of patients with pancreas or breast adenocarcinoma. J Immunol. (2002) 169:2756–61. 10.4049/jimmunol.169.5.2756 12193750

[10] Plitas G Konopacki C Wu K Bos PD Morrow M Putintseva EV. Regulatory T cells exhibit distinct features in human breast cancer. Immunity (2016) 45:1122–34. 10.1016/j.immuni.2016.10.032 27851913 PMC5134901

[11] Kulkarni MM. Digital multiplexed gene expression analysis using the NanoString nCounter system. Curr Protoc Mol Biol. (2011) Chapter 25:Unit25B 10. 10.1002/0471142727.mb25b10s94 21472696

[12] Cesano A. nCounter((R)) PanCancer Immune Profiling Panel (NanoString Technologies, Inc., Seattle, WA). J Immunother Cancer (2015) 3:42. 10.1186/s40425-015-0088-7 26674611 PMC4678588

[13] Strauss L Bergmann C Szczepanski MJ Lang S Kirkwood JM Whiteside TL. Expression of ICOS on human melanoma-infiltrating CD4+CD25highFoxp3+ T regulatory cells: implications and impact on tumor-mediated immune suppression. J Immunol. (2008) 180:2967–80. 10.4049/jimmunol.180.5.2967 18292519

[14] Kotsakis A Harasymczuk M Schilling B Georgoulias V Argiris A Whiteside TL. Myeloid-derived suppressor cell measurements in fresh and cryopreserved blood samples. J Immunol Methods (2012) 381:14–22. 10.1016/j.jim.2012.04.004 22522114 PMC3385927

[15] Konjevic G Jurisic V Spuzic I. Association of NK cell dysfunction with changes in LDH characteristics of peripheral blood lymphocytes (PBL) in breast cancer patients. Breast Cancer Res Treat (2001) 66:255–63. 10.1023/A:1010602822483 11510697

[16] Wijayahadi N Haron MR Stanslas J Yusuf Z. Changes in cellular immunity during chemotherapy for primary breast cancer with anthracycline regimens. J Chemother. (2007) 19:716–23. 10.1179/joc.2007.19.6.716 18230556

[17] Verma R Foster RE Horgan K Mounsey K Nixon H Smalle N. Lymphocyte depletion and repopulation after chemotherapy for primary breast cancer. Breast Cancer Res. (2016) 18:10. 10.1186/s13058-015-0669-x 26810608 PMC4727393

[18] Gyorffy B Lanczky A Eklund AC Denkert C Budczies J Li Q. An online survival analysis tool to rapidly assess the effect of 22,277 genes on breast cancer prognosis using microarray data of 1,809 patients. Breast Cancer Res Treat (2010) 123:725–31. 10.1007/s10549-009-0674-9 20020197

[19] Verma C Eremin JM Robins A Bennett AJ Cowley GP El-Sheemy MA. Abnormal T regulatory cells (Tregs: FOXP3+, CTLA-4+), myeloid-derived suppressor cells (MDSCs: monocytic, granulocytic) and polarised T helper cell profiles (Th1, Th2, Th17) in women with large and locally advanced breast cancers undergoing neoadjuvant chemotherapy (NAC) and surgery: failure of abolition of abnormal treg profile with treatment and correlation of treg levels with pathological response to NAC. J Transl Med. (2013) 11:16. 10.1186/1479-5876-11-16 23320561 PMC3618083

[20] Wang J Yang J. Identification of CD4(+)CD25(+)CD127(−) regulatory T cells and CD14(+)HLA(−)DR(−)/low myeloid-derived suppressor cells and their roles in the prognosis of breast cancer. Biomed Rep. (2016) 5:208–12. 10.3892/br.2016.694 27446543 PMC4950717

[21] Ito T Hanabuchi S Wang YH Park WR Arima K Bover L. Two functional subsets of FOXP3+ regulatory T cells in human thymus and periphery. Immunity (2008) 28:870–80. 10.1016/j.immuni.2008.03.018 18513999 PMC2709453

[22] Mohr A Malhotra R Mayer G Gorochov G Miyara M. Human FOXP3(+) T regulatory cell heterogeneity. Clin Transl Immunol. (2018) 7:e1005. 10.1002/cti2.1005 29484183 PMC5822410

[23] Jurisic V Colovic M. Correlation of sera TNF-alpha with percentage of bone marrow plasma cells, LDH, beta2-microglobulin, and clinical stage in multiple myeloma. Med Oncol. (2002) 19:133–9. 10.1385/MO:19:3:133 12482123

[24] Miyara M Yoshioka Y Kitoh A Shima T Wing K Niwa A. Functional delineation and differentiation dynamics of human CD4+ T cells expressing the FoxP3 transcription factor. Immunity (2009) 30:899–911. 10.1016/j.immuni.2009.03.019 19464196

[25] Kordasti S Costantini B Seidl T Perez Abellan P Martinez Llordella M Mclornan D. Deep phenotyping of Tregs identifies an immune signature for idiopathic aplastic anemia and predicts response to treatment. Blood (2016) 128:1193–205. 10.1182/blood-2016-03-703702 27281795 PMC5009512

[26] Speigl L Burow H Bailur JK Janssen N Walter CB Pawelec G. CD14+ HLA-DR-/low MDSCs are elevated in the periphery of early-stage breast cancer patients and suppress autologous T cell proliferation. Breast Cancer Res Treat (2018) 168:401–11. 10.1007/s10549-017-4594-9 29230664

[27] Bailur JK Gueckel B Derhovanessian E Pawelec G. Presence of circulating Her2-reactive CD8 + T-cells is associated with lower frequencies of myeloid-derived suppressor cells and regulatory T cells, and better survival in older breast cancer patients. Breast Cancer Res. (2015) 17:34. 10.1186/s13058-015-0541-z 25849846 PMC4377034

[28] Diaz-Montero CM Salem ML Nishimura MI Garrett-Mayer E Cole DJ Montero AJ. Increased circulating myeloid-derived suppressor cells correlate with clinical cancer stage, metastatic tumor burden, and doxorubicin-cyclophosphamide chemotherapy. Cancer Immunol Immunother. (2009) 58:49–59. 10.1007/s00262-008-0523-4 18446337 PMC3401888

[29] Gonda K Shibata M Ohtake T Matsumoto Y Tachibana K Abe N. Myeloid-derived suppressor cells are increased and correlated with type 2 immune responses, malnutrition, inflammation, and poor prognosis in patients with breast cancer. Oncol Lett. (2017) 14:1766–74. 10.3892/ol.2017.6305 28789407 PMC5529875

[30] Weiss JM Back TC Scarzello AJ Subleski JJ Hall VL Stauffer JK. Successful immunotherapy with IL-2/anti-CD40 induces the chemokine-mediated mitigation of an immunosuppressive tumor microenvironment. Proc Natl Acad Sci USA. (2009) 106:19455–60. 10.1073/pnas.0909474106 19892741 PMC2773732

[31] Rutella S Filippini P Bertaina V Li Pira G Altomare L Ceccarelli S. Mobilization of healthy donors with plerixafor affects the cellular composition of T-cell receptor (TCR)-alphabeta/CD19-depleted haploidentical stem cell grafts. J Transl Med. (2014) 12:240. 10.1186/s12967-014-0240-z 25179788 PMC4158047

[32] Bailur JK Pawelec G Hatse S Brouwers B Smeets A Neven P. Immune profiles of elderly breast cancer patients are altered by chemotherapy and relate to clinical frailty. Breast Cancer Res. (2017) 19:20. 10.1186/s13058-017-0813-x 28241844 PMC5330012

[33] Feng AL Zhu JK Sun JT Yang MX Neckenig MR Wang XW. CD16+ monocytes in breast cancer patients: expanded by monocyte chemoattractant protein-1 and may be useful for early diagnosis. Clin Exp Immunol. (2011) 164:57–65. 10.1111/j.1365-2249.2011.04321.x 21361908 PMC3074217

[34] Dewan MZ Terunuma H Takada M Tanaka Y Abe H Sata T. Role of natural killer cells in hormone-independent rapid tumor formation and spontaneous metastasis of breast cancer cells *in vivo*. Breast Cancer Res Treat (2007) 104:267–75. 10.1007/s10549-006-9416-4 17066321

[35] Imai K Matsuyama S Miyake S Suga K Nakachi K. Natural cytotoxic activity of peripheral-blood lymphocytes and cancer incidence: an 11-year follow-up study of a general population. Lancet (2000) 356:1795–9. 10.1016/S0140-6736(00)03231-1 11117911

[36] Mamessier E Sylvain A Thibult ML Houvenaeghel G Jacquemier J Castellano R. Human breast cancer cells enhance self tolerance by promoting evasion from NK cell antitumor immunity. J Clin Invest. (2011) 121:3609–22. 10.1172/JCI45816 21841316 PMC3171102

[37] Savas P Virassamy B Ye C Salim A Mintoff CP Caramia F. Single-cell profiling of breast cancer T cells reveals a tissue-resident memory subset associated with improved prognosis. Nat Med. (2018) 24:986–93. 10.1038/s41591-018-0078-7 29942092

