# Supplementary material for: Immune-Phenotyping and Transcriptomic Profiling of Peripheral Blood Mononuclear Cells From Patients With Breast Cancer: Identification of a 3 Gene Signature Which Predicts Relapse of Triple Negative Breast Cancer
Source: Front Immunol. 2018 Sep 11;9:2028. doi: 10.3389/fimmu.2018.02028 (PMC6141692; doi:10.3389/fimmu.2018.02028)
Supplement: Supplementary Figure 1 — Despite the significant loss of gMDSCs after defrosting, significant differences in the percentage of CD15+ cells within the DRnegCD11b+CD33+ population between healthy controls and patients is retained. PBMCs from patients with breast cancer and individuals with no-known disease were rapidly defrosted, allowed to rest for 2 h at 37°C, washed and then incubated with an Fc blocking reagent before being stained with a cocktail of mAbs reactive with cell surface antigens. Alternatively, freshly isolated PBMCs from a proportion of patients and healthy individuals were stained using a cocktail of mAbs that define MDSCs. Data were acquired using a Beckman Coulter Gallios™ flow cytometer and analyzed using Beckman Coulter Kaluza™ software. A two-tailed Mann Whitney test was performed to assess differences between patients with cancer and their corresponding controls, and a Wilcoxon matched-pairs two-tailed test was used to assess the influence of chemotherapy. A Kruskal-Wallis test was used to assess the significance of any differences in the measured parameters between the different disease stages, and individuals with no-known disease (*P < 0.05; **P < 0.005; ***P < 0.0005). The majority of granulocytic MDSCs (DRnegCD11b+CD33+CD15+) did not survive the freezing/defrosting procedure (Figure 1A). However, the significantly higher percentage of CD15+ cells within the DRnegCD11b+CD33+ population in the PBMCs from patients with breast cancer (compared with PBMCs from age-matched controls) remained apparent in the defrosted PBMCs (Figure 1B), with a significantly higher proportion being apparent in the early stages of disease (Stage1) (Figure 1C). [file Data_Sheet_1.PDF]

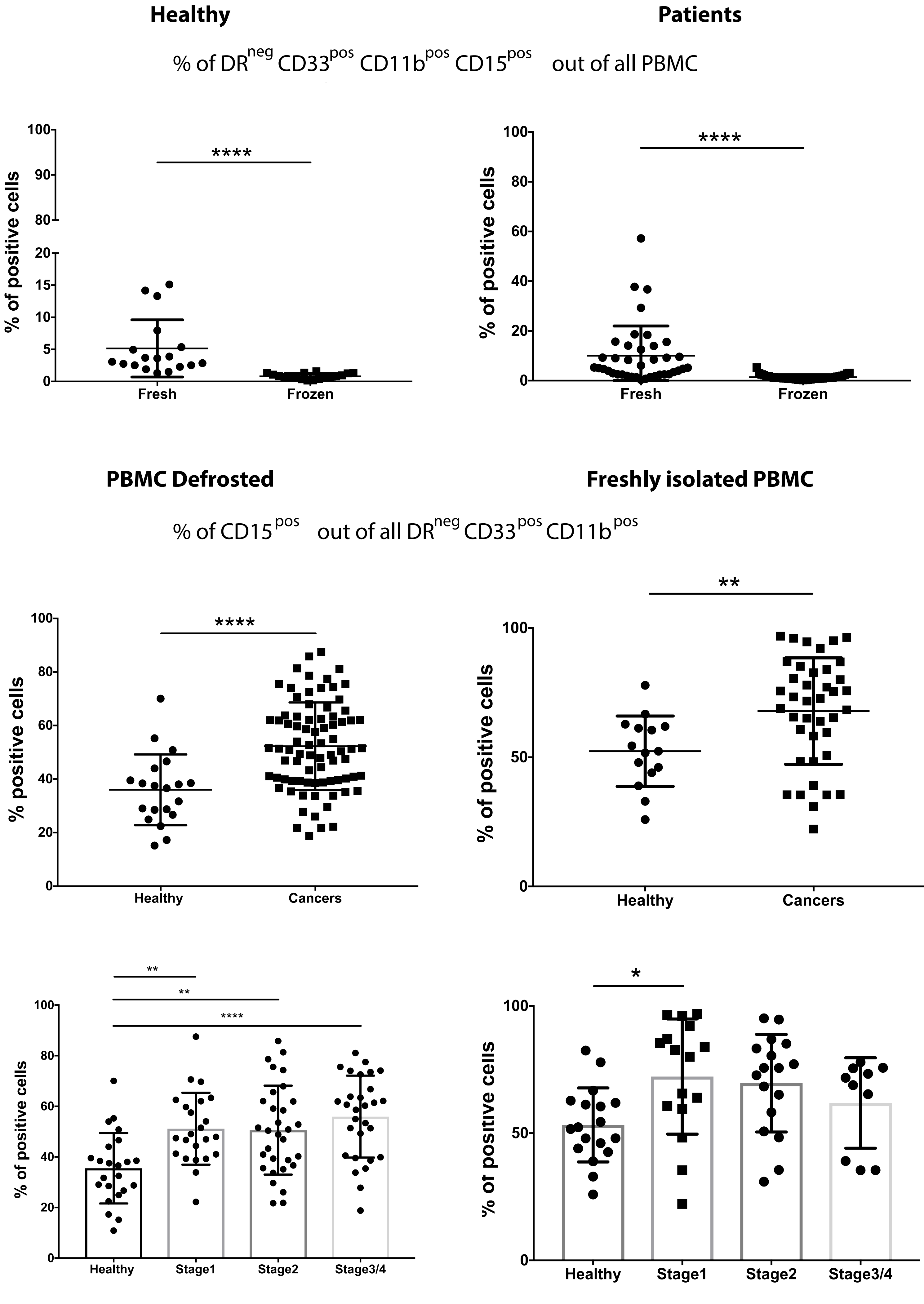

Suppl. Figure 1
