# Supplementary figures and images for: Immune-Phenotyping and Transcriptomic Profiling of Peripheral Blood Mononuclear Cells From Patients With Breast Cancer: Identification of a 3 Gene Signature Which Predicts Relapse of Triple Negative Breast Cancer
Source: Front Immunol. 2018 Sep 11;9:2028. doi: 10.3389/fimmu.2018.02028 (PMC6141692; doi:10.3389/fimmu.2018.02028)

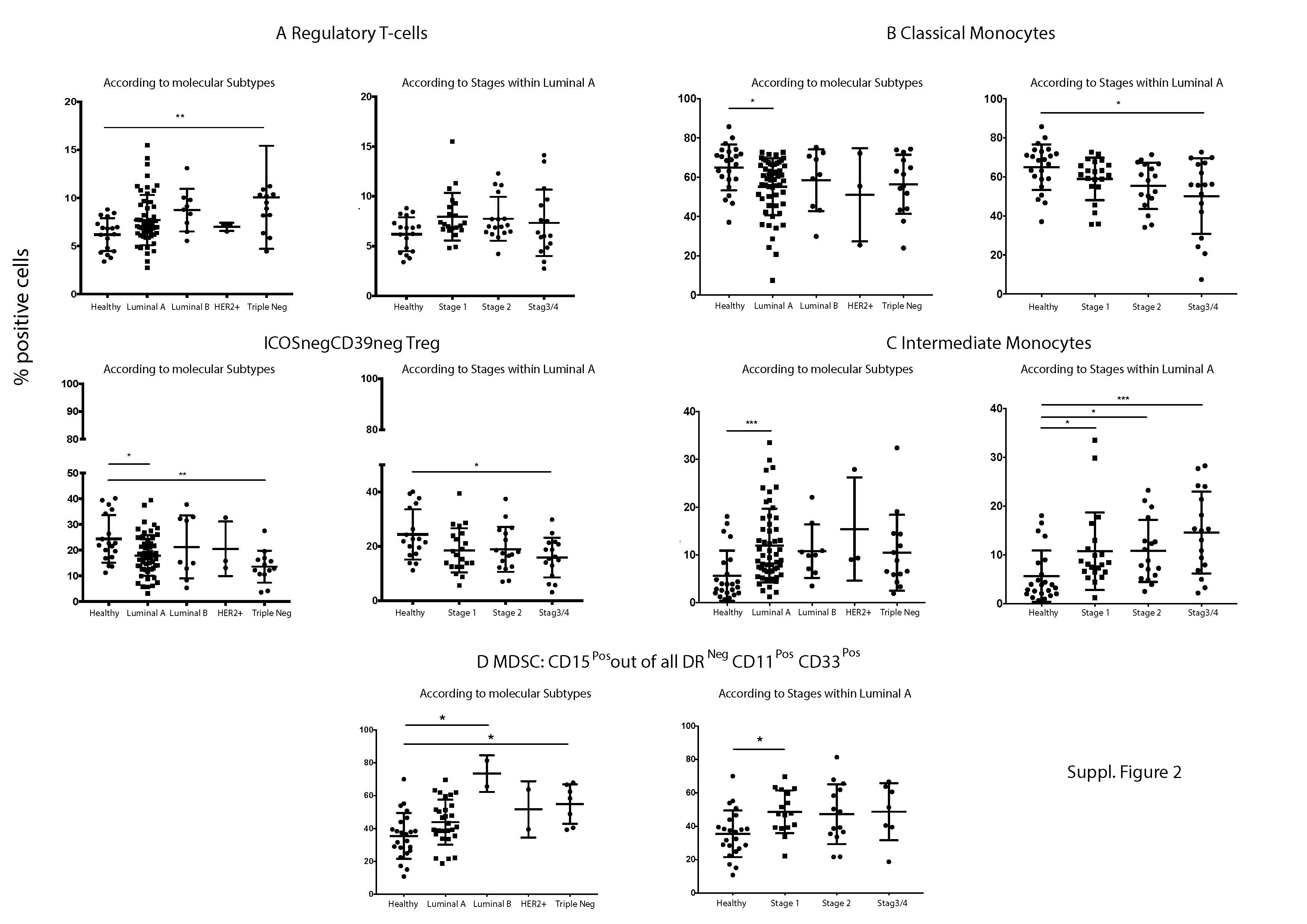

Supplement: Supplementary Figure 2 — NK cell subsets in the periphery of healthy controls and patients with breast cancer (A), and in patients with breast cancer after one round of chemotherapy (B). Similar proportions of CD56dimCD16+ and CD56brightCD16− NK cells were present in patients with breast cancer and healthy controls (A). Chemotherapy had no effect on the intensity of CD16 expression (B). [file Image_2.JPEG]

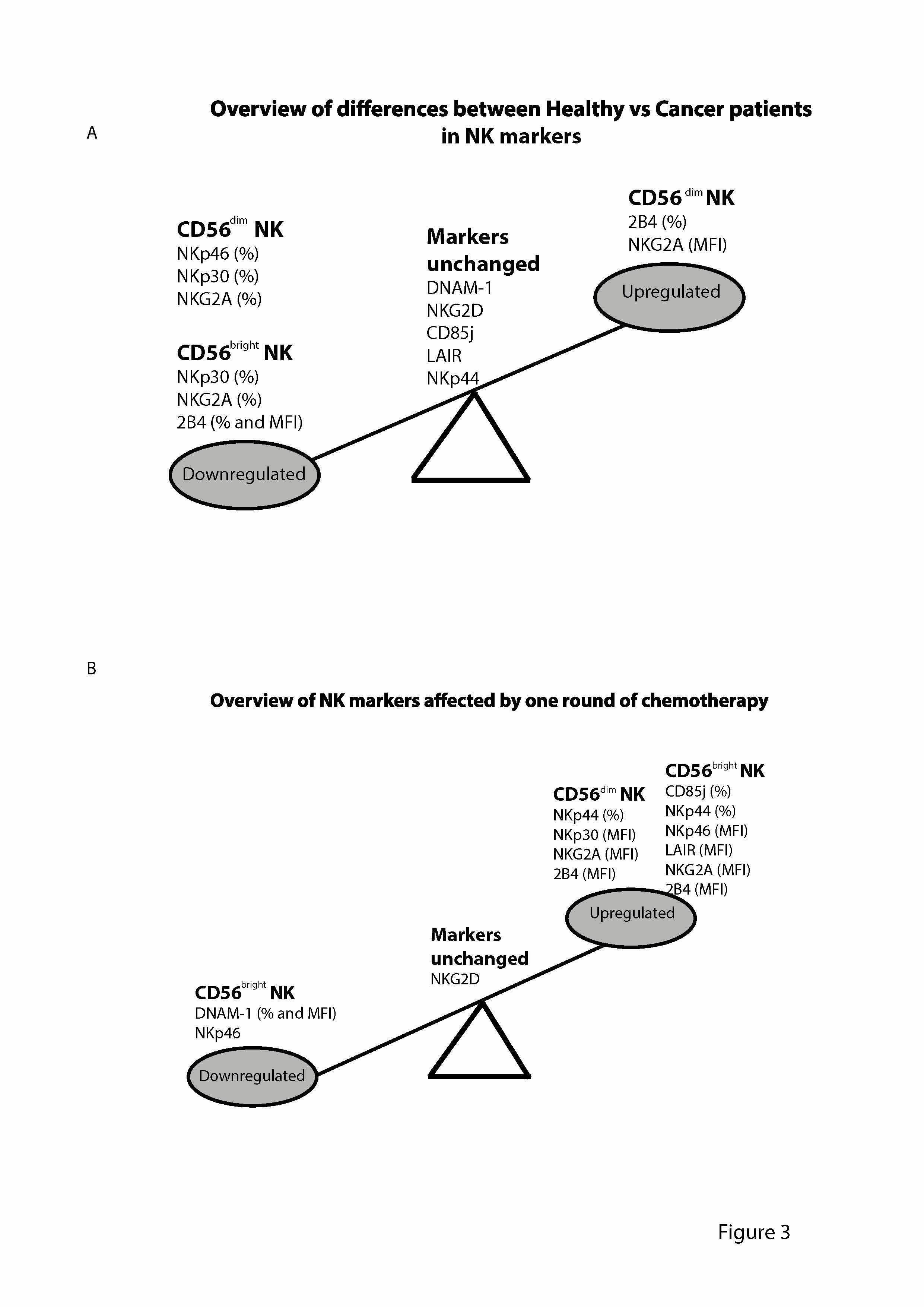

Supplement: Supplementary Figure 3 — The percentage of immunosuppressive immune cells increases with tumor stage. PBMCs from patients with breast cancer and individuals with no-known disease were rapidly defrosted, allowed to rest for 2 h at 37°C, washed and then incubated with an Fc blocking reagent before being stained with a cocktail of mAbs reactive with cell surface antigens. Data were acquired using a Beckman Coulter Gallios™ flow cytometer and analyzed using Beckman Coulter Kaluza™ software. A two-tailed Mann–Whitney test was performed to assess differences between patients with cancer and their corresponding controls, and a Wilcoxon matched-pairs two-tailed test was used to assess the influence of chemotherapy. A Kruskal–Wallis test was used to assess the significance of any differences in the measured parameters between the different disease stages, and individuals with no-known disease (*P < 0.05; **P < 0.005; ***P < 0.0005). Significant differences in the percentage of (A) Treg, (B) classical (CD14++CD16neg), or (C) intermediate (CD14++/+CD16+) monocyte subtypes and the percentage of (D) MDSCs (DRnegCD11b+CD33+CD15+) were primarily found between healthy controls and patients with cancer, rather than between difference molecular subtypes of cancer. However, the majority of patients had luminal disease. Consequently, a subsequent analysis was performed on patients diagnosed with Luminal A only and those with a higher stage disease tended to have less Treg CD39+/ICOS+, less classical monocytes, and more intermediate monocytes and more gMDSCs. [file Image_5.JPEG]

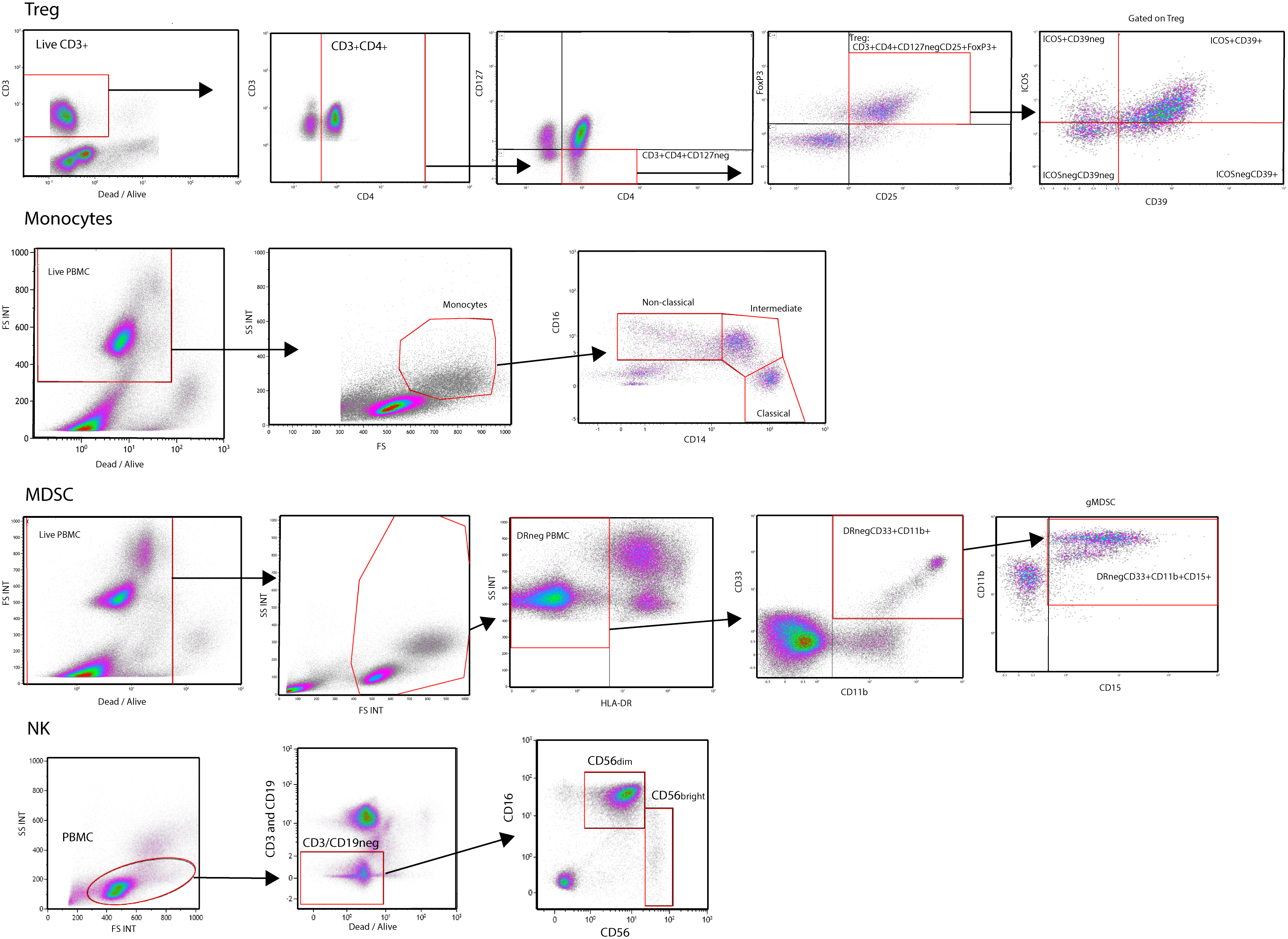

Supplement: Supplementary file 8 [file Image_1.TIF]
