# Supplementary material for: Immune-Phenotyping and Transcriptomic Profiling of Peripheral Blood Mononuclear Cells From Patients With Breast Cancer: Identification of a 3 Gene Signature Which Predicts Relapse of Triple Negative Breast Cancer
Source: Front Immunol. 2018 Sep 11;9:2028. doi: 10.3389/fimmu.2018.02028 (PMC6141692; doi:10.3389/fimmu.2018.02028)
Supplement: Supplementary Table 1 — Patient demographics and summary of flow cytometric analysis. [file Table_1.pdf]

Patients code	*Phenotyping (Pre-treatment)*						*Immunophenotyping of Patients Pre-Chemo therapy*						*Immunophenotyping of Patients Post-Chemo therapy*			
	**Patient Age At Diagnosis**	**TNM**	**ER Status**	**PR Status**	**Her2 Status**	**Pre-Chemo Phenotype Status**										
	**Years**	**As per key**	**0 = -ve, 1 = +ve**	**0 = -ve, 1 = +ve**	**0 = -ve, 1 = +ve**	**Luminal A, Luminal B, Her2+ve, Triple-ve**	OVIS	Treg	MDSC	NK	Monocytes	NanoString	Treg	MDSC	NK	Monocytes
TRG002257	39	T1N1M0	0	0	0	Triple -ve	**N**	**N**	**N**	**N**	**N**	Y				
TRG002262	63	T3N1M0	1	1	0	Luminal A	**N**	Y	Y	**N**	Y		Y	Y	**N**	Y
TRG002263	42	T3N1M0	1	0	0	Luminal A	**N**	**N**	Y	**N**	**N**		**N**	Y	**N**	Y
TRG002264	40	T3N1M0	1	1	0	Luminal A	**N**	Y	Y	**N**	Y		Y	Y	**N**	Y
TRG002269	51	T2N1M0	1	0	1	Luminal B	**N**	**N**	**N**	**N**	**N**		**N**	**N**	**N**	Y
TRG002271	66	T4N1M0	0	0	0	Triple -ve	**N**	**N**	Y	**N**	**N**	Y	**N**	Y	**N**	Y
TRG002276	47	T2N1M0	0	0	0	Triple -ve	**N**	**N**	**N**	**N**	**N**	Y				
TRG002288	36	T2N0M0	1	0	0	Luminal A	**N**	**N**	Y	**N**	**N**		**N**	Y	**N**	Y
TRG002270	61	T1N1M0	1	0	0	Luminal A	**N**	Y	Y	**N**	Y		Y	Y	**N**	Y
TRG002279	47	T4N1M0	1	1	0	Luminal A	**N**	Y	Y	**N**	Y		Y	Y	**N**	Y
TRG002285	57	T2N0M0	1	0	0	Luminal A	**N**	Y	Y	**N**	Y		Y	Y	**N**	Y
TRG002286	54	T2N0M0	0	0	0	Triple -ve	**N**	Y	Y	**N**	Y	Y	Y	Y	**N**	Y
TRG002290	63	T2N0M0	0	0	0	Triple -ve	**N**	Y	Y	**N**	**N**	Y	N	Y	**N**	Y
TRG002293	45	T3N0M0	1	1	0	Luminal A	**N**	Y	Y	Y	Y		Y	Y	Y	Y
TRG002295	35	T2N1M0	1	1	0	Luminal A	**N**	Y	Y	**N**	Y		Y	Y	**N**	Y
TRG002298	49	T2N1M0	1	1	0	Luminal A	**N**	Y	Y	**N**	Y		Y	Y	**N**	Y
TRG002301	56	T4N1M0	1	1	0	Luminal A	**N**	Y	Y	Y	Y		Y	Y	Y	Y
TRG002306	63	T2N1M0	1	1	0	Luminal A	Y	Y	Y	**N**	Y		Y	Y	**N**	Y
TRG002311	51	T4N1M0	1	0	0	Luminal A	Y	Y	Y	Y	Y		Y	Y	Y	Y
TRG002316	68	T4N1M0	0	0	1	Her2 +ve	Y	Y	Y	Y	Y		Y	Y	Y	Y
TRG002317	39	T4N1M0	0	0	0	Triple -ve	Y	Y	Y	Y	Y	Y	Y	Y	Y	Y
TRG002318	49	T2N1M0	1	0	1	Luminal B	Y	Y	Y	Y	Y		Y	Y	Y	Y
TRG002320	47	T2N1M0	1	0	1	Luminal B	Y	Y	Y	Y	Y		Y	Y	Y	Y
TRG002325	47	T4N1M0	1	1	0	Luminal A	Y	Y	Y	Y	Y		Y	Y	Y	Y

TRG002326	45	T1N1M0	1	0	0	Luminal A	Y	Y	Y	Y	Y		Y	Y	Y	Y
TRG002327	47	T4N1M0	0	0	0	Triple -ve	**N**	**N**	**N**	**N**	**N**	Y	**N**	**N**	**N**	**N**
TRG002328	64	T4N1M0	1	0	0	Luminal A	Y	Y	Y	Y	Y		Y	Y	Y	Y
TRG002329	56	T3N1M0	1	1	0	Luminal A	Y	Y	Y	Y	Y		Y	Y	Y	Y
TRG002332	35	T4N1M0	1	0	1	Luminal B	Y	Y	Y	**N**	Y		Y	Y	**N**	Y
TRG002334	46	T4N1M0	1	0	1	Luminal B	Y	Y	Y	Y	Y		Y	Y	Y	Y
TRG002335	54	T4N0M0	1	1	0	Luminal A	Y	Y	Y	Y	Y		Y	Y	Y	Y
TRG002338	49	T4N1M0	1	0	1	Luminal B	Y	Y	Y	**N**	Y		Y	Y	**N**	Y
TRG002343	47	T3N1M0	1	1	0	Luminal A	Y	**N**	Y	Y	**N**		**N**	Y	Y	Y
TRG002344	47	T2N1M0	1	1	0	Luminal A	Y	Y	Y	Y	Y		Y	Y	Y	Y
TRG002346	41	T2N1M0	1	1	1	Luminal B	Y	Y	Y	Y	Y		Y	Y	Y	Y
TRG002353	59	T4N1M0	0	0	0	Triple -ve	Y	Y	Y	Y	Y		Y	Y	Y	Y
TRG002354	60	T2N1M0	0	0	0	Triple -ve	Y	Y	Y	Y	Y		Y	Y	Y	Y
TRG002355	73	T2N1M0	0	0	0	Triple -ve	Y	Y	Y	**N**	Y	Y	Y	Y	**N**	Y
TRG002358	72	T2N1M0	0	0	0	Triple -ve	Y	Y	Y	**N**	Y	Y	Y	Y	**N**	Y
TRG002360	67	T4N1M0	0	0	0	Triple -ve	Y	Y	Y	Y	Y		Y	Y	Y	Y
TRG002361	42	T2N1M0	1	0	0	Luminal A	Y	Y	Y	**N**	Y		Y	Y	**N**	Y
TRG002366	60	T4N1M0	1	1	0	Luminal A	Y	Y	Y	Y	Y		Y	Y	Y	Y
TRG002367	40	T2N1M0	1	0	0	Luminal A	Y	Y	Y	Y	Y		Y	Y	Y	Y
TRG002373	45	T4N1M0	0	0	1	Her2 +ve	Y	Y	Y	Y	Y		Y	Y	Y	Y
TRG002375	44	T2N1M0	1	1	0	Luminal A	Y	Y	**N**	Y	Y		Y	**N**	Y	Y
TRG002379	28	T2N0M0	0	0	0	Triple -ve	Y	**N**	**N**	**N**	**N**	Y				
TRG002383	46	T3N1M0	1	1	0	Luminal A	Y	Y	**N**	Y	Y		Y	**N**	Y	Y
TRG002384	62	T4N0M0	1	1	0	Luminal A	Y	Y	Y	Y	Y		Y	Y	Y	Y
TRG002387	64	T4N0M0	0	0	0	Triple -ve	Y	Y	Y	Y	Y		Y	Y	Y	Y
TRG002390	40	T4N1M0	1	1	0	Luminal A	Y	Y	Y	Y	Y		Y	Y	Y	Y
TRG002391	52	T2N0M0	1	1	1	Luminal B	Y	Y	Y	Y	Y		Y	Y	Y	Y
TRG002392	56	T3N1M0	0	0	1	Her2 +ve	Y	Y	Y	Y	Y		Y	Y	Y	Y
TRG002397	60	T2N1M0	1	1	0	Luminal A	Y	Y	**N**	Y	Y		Y	Y	Y	Y

TRG002399	48	T2N1M0	1	1	1	Luminal B	Y	Y	Y	Y	Y		Y	Y	Y	Y
ACP-0001	54	T1cN1M0	1	1	0	Luminal A	Y	Y	Y	Y	Y					
ACP-0002	83	T3N1M0	1	1	0	Luminal A	Y	Y	Y	Y	Y					
ACP-0003	66	T1aN0M0	1	1	0	Luminal A	Y	Y	Y	Y	Y					
ACP-0004	82	T2N1M0	1	1	0	Luminal A	Y	Y	Y	Y	Y					
ACP-0005	82	T2N0M0	1	1	0	Luminal A	Y	Y	Y	Y	Y					
ACP-0006	76	T1cN0M0	1	1	0	Luminal A	Y	Y	Y	Y	Y					
ACP-0007	39	T1cN0M0	1	1	1	Luminal B	Y	Y	Y	Y	Y					
ACP-0008	52	T1bN0M0	1	1	0	Luminal A	Y	Y	Y	Y	Y	Y				
ACP-0009	41	T2N0M0	1	1	0	Luminal A	Y	Y	Y	Y	Y					
ACP-0010	42	T1cN0M0	1	1	0	Luminal A	Y	Y	Y	Y	Y	Y				
ACP-0011	76	T2N0M0	1	1	0	Luminal A	Y	Y	Y	Y	Y					
ACP-0012	65	T1cN0M0	1	1	0	Luminal A	Y	Y	Y	Y	Y					
ACP-0013	49	T2N0M0	0	0	0	Triple -ve	Y	Y	Y	Y	Y	Y				
ACP-0014	42	T2N1M0	1	1	0	Luminal A	Y	Y	Y	Y	Y					
ACP-0015	66	T2N0M0	1	1	0	Luminal A	Y	Y	Y	Y	Y					
ACP-0016	48	T1cN0M0	1	1	0	Luminal A	Y	Y	Y	Y	Y					
ACP-0017	53	T1aN0M0	1	1	0	Luminal A	Y	Y	Y	Y	Y	Y				
ACP-0018	72	T2N0M0	0	0	0	Triple -ve	Y	Y	Y	Y	Y	Y				
ACP-0019	37	T2N0M0	1	1	0	Luminal A	Y	Y	Y	Y	Y					
ACP-0020	66	T3N0M0	1	1	0	Luminal A	Y	Y	Y	Y	Y					
ACP-0021	65	T1cN0M0	1	1	0	Luminal A	Y	Y	Y	Y	Y					
ACP-0022	54	T1bN0M0	1	1	0	Luminal A	Y	Y	Y	Y	Y					
ACP-0023	77	T2N0M0	1	1	0	Luminal A	Y	Y	Y	Y	Y					
ACP-0025	89	T2N0M0	0	0	0	Triple -ve	Y	Y	Y	Y	Y	Y				
ACP-0026	79	T1cN0M0	0	0	0	Triple -ve	Y	Y	Y	Y	Y	Y				
ACP-0027	65	T1cN1M0	1	1	0	Luminal A	Y	Y	Y	Y	Y					
ACP-0028	63	T1cN0M0	1	1	0	Luminal A	Y	Y	Y	Y	Y					
ACP-0029	61	T1bN1M0	1	1	0	Luminal A	Y	Y	Y	Y	Y	Y				
ACP-0030	52	T1bN0M0	1	0	0	Luminal A	Y	Y	Y	Y	Y	Y				

ACP-0031	69	T1bN0M0	1	1	0	Luminal A	Y	Y	Y	Y	Y	Y
ACP-0032	61	T1cN3M1	1	1	0	Luminal A	Y	Y	Y	Y	Y	
ACP-0033	61	T1bN0M0	1	1	0	Luminal A	Y	Y	Y	Y	Y	Y
ACP-0034	63	T1cN1M0	1	1	0	Luminal A	Y	Y	Y	Y	Y	Y
ACP-0035	68	T1bN0M0	1	1	0	Luminal A	Y	Y	Y	Y	Y	Y
Healthy Donors												
BrCa0001	43						Y	Y	Y	Y	Y	
BrCa0002	35						Y	Y	Y	Y	Y	
BrCa0003	48						Y	Y	Y	Y	Y	
BrCa0004	46						Y	Y	Y	Y	Y	
BrCa0006	38						Y	Y	Y	Y	Y	
BrCa0007	45						Y	Y	Y	Y	Y	
BrCa0008	43						Y	**N**	Y	Y	Y	
BrCa0009	51						Y	**N**	Y	Y	Y	
BrCa0010	42						Y	Y	Y	Y	Y	
BrCa0012	41						Y	Y	Y	Y	Y	
BrCa0013	44						Y	Y	Y	Y	Y	
BrCa0015	46						Y	Y	Y	Y	Y	
BrCa0016	37						Y	Y	Y	Y	Y	
BrCa0017	53						Y	Y	Y	Y	Y	
BrCa0019	52						Y	Y	Y	Y	Y	
BrCa0020	56						Y	Y	Y	Y	Y	
BrCa0022	44						Y	**N**	Y	**N**	Y	
BrCa0023	65						Y	Y	Y	Y	Y	
BrCa0024	59						Y	**N**	Y	**N**	Y	
BrCa0025	58						Y	Y	Y	Y	Y	
BrCa0026	57						Y	**N**	Y	**N**	Y	
BrCa0027	65						Y	Y	Y	Y	Y	
BrCa0028	61						Y	Y	Y	Y	Y	
