# Supplementary material for: Immune-Phenotyping and Transcriptomic Profiling of Peripheral Blood Mononuclear Cells From Patients With Breast Cancer: Identification of a 3 Gene Signature Which Predicts Relapse of Triple Negative Breast Cancer
Source: Front Immunol. 2018 Sep 11;9:2028. doi: 10.3389/fimmu.2018.02028 (PMC6141692; doi:10.3389/fimmu.2018.02028)
Supplement: Supplementary Table 2 — Antibody panels used in this study. [file Table_2.pdf]

Antibody	Company	Ab used in Panel
CD8-FITC	BioLegend	OVIS
CD19-PE	BioLegend	OVIS
CD3-PE/Cy7	BioLegend	OVIS
CD56-PE/Cy5	BioLegend	OVIS
CD14-Alexa Fluor700	BioLegend	OVIS
CD4-Krome orange	Beckman Coulter	OVIS, Treg
CD45-Pacific Blue	Beckman Coulter	OVIS
HLA-DR-FITC	BioLegend	MDSC (Fresh)
HLA-DR-APC-Cy7	BioLegend	MDSC (Frozen)
CD11b-PE	BioLegend	MDSC (Frozen)
Arginase 1-Alexa Fluor 700	R&D System	MDSC (Fresh and Frozen)
CD33-PE/C5	BioLegend	MDSC (Fresh and Frozen)
CD15-PE/Cy7	BioLegend	MDSC (Fresh and Frozen)
LIN-APC	BioLegend	MDSC (Fresh)
CD45-Pacific Blue	Beckman Coulter	MDSC (Fresh)
CD14-APC	BioLegend	MDSC (Frozen), Monocytes
CD8 APC/Cy7	BioLegend	NK
CD56-ECD (Texas Red)	Beckman Coulter	NK
CD16-PerCP/Cy5.5	BioLegend	NK
CD3-Alexa Fluor 700	BioLegend	NK
CD19-Alexa Fluor 700	BioLegend	NK
NKp46-PE/Cy7	BioLegend	NK
DNMA1 FITC	BioLegend	NK
NKp30 Alexa Fluor 647	BioLegend	NK
NKp44-Alexa Fluor 647	BioLegend	NK
2B4-FITC	BioLegend	NK
CD85j-FITC	Miltenyi Biotec	NK
NKG2D-PE	eBioscience	NK
CD56-ECD	Beckman Coulter	NK
LAIR-PE	Pharmingen	NK
NKG2A PE/Cy7	Beckman Coulter	NK
CD16-Krome orange	Beckman Coulter	Monocytes
ICOS-PE	BioLegend	Treg
CD3-ECD	Beckman Coulter	Treg
CD25-PE/CyC5.5	Beckman Coulter	Treg
CD8-Alexa Fluor 700	Pharmingen	Treg
CD39-PE/Cy7	eBioscience	Treg
CD127-APC/eFluor 780	eBioscience	Treg
FoxP3-APC	eBioscience	Treg
Violet Fixable Live/Dead stain	InVitrogen	All panels
