# Supplementary material for: Immune-Phenotyping and Transcriptomic Profiling of Peripheral Blood Mononuclear Cells From Patients With Breast Cancer: Identification of a 3 Gene Signature Which Predicts Relapse of Triple Negative Breast Cancer
Source: Front Immunol. 2018 Sep 11;9:2028. doi: 10.3389/fimmu.2018.02028 (PMC6141692; doi:10.3389/fimmu.2018.02028)
Supplement: Supplementary Table 3 — Identification of an immune gene signature predicting high risk of relapse and poor survival in TNBC. The 20 most differentially regulated genes identified by the nSolver software package (Nanostring Technologies) were assessed by Metacore, the results of which are shown here (Table 3). [file Table_3.pdf]

Diseases	*Total*	*pValues*	*Min FDR*	*p-Value*	*FDR*	*In Data*	*Network objects from Active Data*
Inflammation	966	5.716E-12	5.356E-09	5.716E-12	5.356E-09	10	CLEC4C, Galpha(q)-specific peptide GPCRs, Galpha(i)-specific peptide GPCRs, IFNGR1, IL1RAP, Thrombospondin 1, TXNIP (VDUP1), CXCR4, TLR7, CD163
Connective Tissue Diseases	2791	2.458E-10	9.309E-08	2.458E-10	9.309E-08	12	MFGE8, IL1R2, CLEC4C, Galpha(q)-specific peptide GPCRs, Galpha(i)-specific peptide GPCRs, IFNGR1, Thrombospondin 1, TXNIP (VDUP1), CXCR4, LTB, TLR7, CD163
Pathologic Processes	2837	2.981E-10	9.309E-08	2.981E-10	9.309E-08	12	MFGE8, CLEC4C, Galpha(q)-specific peptide GPCRs, Galpha(i)-specific peptide GPCRs, IFNGR1, IL1RAP, Thrombospondin 1, TXNIP (VDUP1), CXCR4, FLT3, TLR7, CD163
Joint Diseases	2154	4.977E-10	1.024E-07	4.977E-10	1.024E-07	11	IL1R2, CLEC4C, Galpha(q)-specific peptide GPCRs, Galpha(i)-specific peptide GPCRs, IFNGR1, Thrombospondin 1, TXNIP (VDUP1), CXCR4, LTB, TLR7, CD163
Whim Syndrome	4	5.463E-10	1.024E-07	5.463E-10	1.024E-07	3	Galpha(q)-specific peptide GPCRs, Galpha(i)-specific peptide GPCRs, CXCR4
Arthritis, Rheumatoid	1888	4.134E-09	6.392E-07	4.134E-09	6.392E-07	10	IL1R2, CLEC4C, Galpha(q)-specific peptide GPCRs, Galpha(i)-specific peptide GPCRs, IFNGR1, TXNIP (VDUP1), CXCR4, LTB, TLR7, CD163
Rare Immune System Diseases	7	4.775E-09	6.392E-07	4.775E-09	6.392E-07	3	Galpha(q)-specific peptide GPCRs, Galpha(i)-specific peptide GPCRs, CXCR4
Gastroenteritis	1360	5.939E-09	6.663E-07	5.939E-09	6.663E-07	9	MFGE8, IL1R2, Galpha(q)-specific peptide GPCRs, Galpha(i)-specific peptide GPCRs, IFNGR1, Thrombospondin 1, TXNIP (VDUP1), CXCR4, TLR7
AIDS Dementia Complex	8	7.638E-09	6.663E-07	7.638E-09	6.663E-07	3	Galpha(q)-specific peptide GPCRs, Galpha(i)-specific peptide GPCRs, CXCR4
Hemangioblastoma	8	7.638E-09	6.663E-07	7.638E-09	6.663E-07	3	Galpha(q)-specific peptide GPCRs, Galpha(i)-specific peptide GPCRs, CXCR4
