# Supplementary material for: Immune-Phenotyping and Transcriptomic Profiling of Peripheral Blood Mononuclear Cells From Patients With Breast Cancer: Identification of a 3 Gene Signature Which Predicts Relapse of Triple Negative Breast Cancer
Source: Front Immunol. 2018 Sep 11;9:2028. doi: 10.3389/fimmu.2018.02028 (PMC6141692; doi:10.3389/fimmu.2018.02028)
Supplement: Supplementary file 7 [file Table_4.PDF]

Patients code	Patient Age at Diagnosis Years	TNM	Grade	Phenotyping (Pre-treatment)				Immunophenotyping of Patients Pre-Chemo therapy						Immunophenotyping of Patients Post-Chemo therapy			
				ER Status	PR Status	Her2 Status	Pre-Chemo Phenotype Status										
				0 = -ve, 1 = +ve	0 = -ve, 1 = +ve	0 = -ve, 1 = +ve	Luminal A or B, Her2+ve, Triple-ve	OVIS	Treg	MDSC	NK	Monocytes	NanoString	Treg	MDSC	NK	Monocytes
TRG03000	39	T1N1M0	1	0	0	0	Triple -ve	N	N	N	N	N	Y	N	N	N	N
TRG03001	63	T3N1M0	3	1	1	0	Luminal A	N	Y	Y	N	Y		Y	Y	N	Y
TRG03002	42	T3N1M0	3	1	0	0	Luminal A	N	N	Y	N	N		N	Y	N	Y
TRG03003	40	T3N1M0	3	1	1	0	Luminal A	N	Y	Y	N	Y		Y	Y	N	Y
TRG03004	51	T2N1M0	2	1	0	1	Luminal B	N	N	N	N	N		N	N	N	Y
TRG03005	61	T1N1M0	1	1	0	0	Luminal A	N	Y	Y	N	Y		Y	Y	N	Y
TRG03006	66	T4N1M0	4	0	0	0	Triple -ve	N	N	Y	N	N	Y	N	Y	N	Y
TRG03007	47	T2N1M0	2	0	0	0	Triple -ve	N	N	N	N	N	Y	N	N	N	N
TRG03008	47	T4N1M0	4	1	1	0	Luminal A	N	Y	Y	N	Y		Y	Y	N	Y
TRG03009	57	T2N0M0	2	1	0	0	Luminal A	N	Y	Y	N	Y		Y	Y	N	Y
TRG03010	54	T2N0M0	2	0	0	0	Triple -ve	N	Y	Y	N	Y	Y	Y	Y	N	Y
TRG03011	36	T2N0M0	2	1	0	0	Luminal A	N	N	Y	N	N		N	Y	N	Y
TRG03012	63	T2N0M0	2	0	0	0	Triple -ve	N	Y	Y	N	N	Y	N	Y	N	Y
TRG03013	45	T3N0M0	3	1	1	0	Luminal A	N	Y	Y	Y	Y		Y	Y	Y	Y
TRG03014	35	T2N1M0	2	1	1	0	Luminal A	N	Y	Y	N	Y		Y	Y	N	Y
TRG03015	49	T2N1M0	2	1	1	0	Luminal A	N	Y	Y	N	Y		Y	Y	N	Y
TRG03016	56	T4N1M0	4	1	1	0	Luminal A	N	Y	Y	Y	Y		Y	Y	Y	Y
TRG03017	63	T2N1M0	2	1	1	0	Luminal A	Y	Y	Y	N	Y		Y	Y	N	Y
TRG03018	51	T4N1M0	4	1	0	0	Luminal A	Y	Y	Y	Y	Y		Y	Y	Y	Y
TRG03019	68	T4N1M0	4	0	0	1	Her2 +ve	Y	Y	Y	Y	Y		Y	Y	Y	Y
TRG03020	39	T4N1M0	4	0	0	0	Triple -ve	Y	Y	Y	Y	Y	Y	Y	Y	Y	Y
TRG03021	49	T2N1M0	2	1	0	1	Luminal B	Y	Y	Y	Y	Y		Y	Y	Y	Y
TRG03022	47	T2N1M0	2	1	0	1	Luminal B	Y	Y	Y	Y	Y		Y	Y	Y	Y
TRG03024	47	T4N1M0	4	1	1	0	Luminal A	N	Y	Y	Y	Y		Y	Y	Y	Y
TRG03025	45	T1N1M0	1	1	0	0	Luminal A	Y	Y	Y	Y	Y		Y	Y	Y	Y
TRG03026	47	T4N1M0	4	0	0	0	Triple -ve	Y	N	N	N	N	Y	N	N	N	N
TRG03027	64	T4N1M0	4	1	0	0	Luminal A	Y	Y	Y	Y	Y		Y	Y	Y	Y
TRG03028	56	T3N1M0	3	1	1	0	Luminal A	Y	Y	Y	Y	Y		Y	Y	Y	Y
TRG03029	35	T4N1M0	4	1	0	1	Luminal B	Y	Y	Y	N	Y		Y	Y	N	Y
TRG03030	46	T4N1M0	4	1	0	1	Luminal B	Y	Y	Y	Y	Y		Y	Y	Y	Y
TRG03031	54	T4N0M0	4	1	1	0	Luminal A	Y	Y	Y	Y	Y		Y	Y	Y	Y
TRG03032	49	T4N1M0	4	1	0	1	Luminal B	Y	Y	Y	N	Y		Y	Y	N	Y
TRG03033	47	T3N1M0	3	1	1	0	Luminal A	Y	N	Y	Y	N		N	Y	Y	Y
TRG03034	47	T2N1M0	2	1	1	0	Luminal A	Y	Y	Y	Y	Y		Y	Y	Y	Y
TRG03000	41	T2N1M0	2	1	1	1	Luminal B	Y	Y	Y	Y	Y		Y	Y	Y	Y
TRG03001	59	T4N1M0	4	0	0	0	Triple -ve	Y	Y	Y	Y	Y		Y	Y	Y	Y

TRG03002	60	T2N1M0	2	0	0	0	Triple -ve	Y	Y	Y	Y	Y		Y	Y	Y	Y
TRG03003	73	T2N1M0	2	0	0	0	Triple -ve	Y	Y	Y	N	Y	Y	Y	Y	N	Y
TRG03004	72	T2N1M0	2	0	0	0	Triple -ve	Y	Y	Y	N	Y	Y	Y	Y	N	Y
TRG03005	67	T4N1M0	4	0	0	0	Triple -ve	Y	Y	Y	Y	Y		Y	Y	Y	Y
TRG03006	42	T2N1M0	2	1	0	0	Luminal A	Y	Y	Y	N	Y		Y	Y	N	Y
TRG03007	60	T4N1M0	4	1	1	0	Luminal A	Y	Y	Y	Y	Y		Y	Y	Y	Y
TRG03008	40	T2N1M0	2	1	0	0	Luminal A	Y	Y	Y	Y	Y		Y	Y	Y	Y
TRG03009	45	T4N1M0	4	0	0	1	Her2 +ve	Y	Y	Y	Y	Y		Y	Y	Y	Y
TRG03010	44	T2N1M0	2	1	1	0	Luminal A	Y	Y	N	Y	Y		Y	N	Y	Y
TRG03011	28	T2N0M0	2	0	0	0	Triple -ve	Y	N	N	N	N	Y	N	N	N	N
TRG03012	46	T3N1M0	3	1	1	0	Luminal A	Y	Y	N	Y	Y		Y	N	Y	Y
TRG03013	62	T4N0M0	4	1	1	0	Luminal A	Y	Y	Y	Y	Y		Y	Y	Y	Y
TRG03014	64	T4N0M0	4	0	0	0	Triple -ve	Y	Y	Y	Y	Y		Y	Y	Y	Y
TRG03015	40	T4N1M0	4	1	1	0	Luminal A	Y	Y	Y	Y	Y		Y	Y	Y	Y
TRG03016	52	T2N0M0	2	1	1	1	Luminal B	Y	Y	Y	Y	Y		Y	Y	Y	Y
TRG03017	56	T3N1M0	3	0	0	1	Her2 +ve	Y	Y	Y	Y	Y		Y	Y	Y	Y
TRG03018	60	T2N1M0	2	1	1	0	Luminal A	Y	Y	N	Y	Y		Y	Y	Y	Y
TRG03019	48	T2N1M0	2	1	1	1	Luminal B	Y	Y	Y	Y	Y		Y	Y	Y	Y
TRG03020	49	T4N1M0	4	1	0	1	Luminal B	Y	Y	Y	Y	Y		N	N	N	N
TRG03021	43	T2N0M0	2	1	1	1	Luminal B	Y	Y	Y	Y	Y		N	N	N	N
TRG03022	54	T1cN1M0	3	1	1	0	Luminal A	Y	Y	Y	Y	Y					
TRG03024	83	T3N1M0	3	1	1	0	Luminal A	Y	Y	Y	Y	Y					
TRG03025	66	T1aN0M0	1	1	1	0	Luminal A	Y	Y	Y	Y	Y					
TRG03026	82	T2N1M0	2	1	1	0	Luminal A	Y	Y	Y	Y	Y					
TRG03027	82	T2N0M0	2	1	1	0	Luminal A	Y	Y	Y	Y	Y					
TRG03028	76	T1cN0M0	2	1	1	0	Luminal A	Y	Y	Y	Y	Y					
TRG03029	39	T1cN0M0	2	1	1	1	Luminal B	Y	Y	Y	Y	Y					
TRG03030	52	T1bN0M0	1	1	1	0	Luminal A	Y	Y	Y	Y	Y	Y				
TRG03031	41	T2N0M0	1	1	1	0	Luminal A	Y	Y	Y	Y	Y					
TRG03032	42	T1cN0M0	2	1	1	0	Luminal A	Y	Y	Y	Y	Y	Y				
TRG03033	76	T2N0M0	2	1	1	0	Luminal A	Y	Y	Y	Y	Y					
TRG03034	65	T1cN0M0	2	1	1	0	Luminal A	Y	Y	Y	Y	Y					
TRG03000	49	T2N0M0	3	0	0	0	Triple -ve	Y	Y	Y	Y	Y	Y				
TRG03001	42	T2N1M0	3	1	1	0	Luminal A	Y	Y	Y	Y	Y					
TRG03002	66	T2N0M0	2	1	1	0	Luminal A	Y	Y	Y	Y	Y					
TRG03003	48	T1cN0M0	2	1	1	0	Luminal A	Y	Y	Y	Y	Y					
TRG03004	53	T1aN0M0	1	1	1	0	Luminal A	Y	Y	Y	Y	Y	Y				
TRG03005	72	T2N0M0	3	0	0	0	Triple -ve	Y	Y	Y	Y	Y	Y				
TRG03006	37	T2N0M0	1	1	1	0	Luminal A	Y	Y	Y	Y	Y					
TRG03007	66	T3N0M0	2	1	1	0	Luminal A	Y	Y	Y	Y	Y					
TRG03008	65	T1cN0M0	2	1	1	0	Luminal A	Y	Y	Y	Y	Y					
TRG03009	54	T1bN0M0	1	1	1	0	Luminal A	Y	Y	Y	Y	Y					
TRG03010	77	T2N0M0	2	1	1	0	Luminal A	Y	Y	Y	Y	Y					
TRG03011	89	T2N0M0	3	0	0	0	Triple -ve	Y	Y	Y	Y	Y	Y				
TRG03012	79	T1cN0M0	2	0	0	0	Triple -ve	Y	Y	Y	Y	Y	Y				

TRG03013	65	T1cN1M0	2	1	1	0	Luminal A	Y	Y	Y	Y	Y	
TRG03014	63	T1cN0M0	2	1	1	0	Luminal A	Y	Y	Y	Y	Y	
TRG03015	61	T1bN1M0	2	1	1	0	Luminal A	Y	Y	Y	Y	Y	Y
TRG03016	52	T1bN0M0	2	1	0	0	Luminal A	N	Y	Y	Y	Y	Y
TRG03017	69	T1bN0M0	1	1	1	0	Luminal A	Y	Y	Y	Y	Y	Y
TRG03018	61	T1cN3M1	3	1	1	0	Luminal A	Y	Y	Y	Y	Y	
TRG03019	61	T1bN0M0	1	1	1	0	Luminal A	Y	Y	Y	Y	Y	Y
TRG03020	63	T1cN1M0	1	1	1	0	Luminal A	Y	Y	Y	Y	Y	Y
TRG03021	68	T1bN0M0	3	1	1	0	Luminal A	Y	Y	Y	Y	Y	Y
Healthy Donors													
BrCa0001	43							Y	Y	Y	Y	Y	
BrCa0002	35							Y	Y	Y	Y	Y	
BrCa0003	48							Y	Y	Y	Y	Y	
BrCa0004	46							Y	Y	Y	Y	Y	
BrCa0006	38							Y	Y	Y	Y	Y	
BrCa0007	45							Y	Y	Y	Y	Y	
BrCa0008	43							Y	N	Y	Y	Y	
BrCa0009	51							Y	N	Y	Y	Y	
BrCa0010	42							Y	Y	Y	Y	Y	
BrCa0012	41							Y	Y	Y	Y	Y	
BrCa0013	44							Y	Y	Y	Y	Y	
BrCa0015	46							Y	Y	Y	Y	Y	
BrCa0016	37							Y	Y	Y	Y	Y	
BrCa0017	53							Y	Y	Y	Y	Y	
BrCa0019	52							Y	Y	Y	Y	Y	
BrCa0020	56							Y	Y	Y	Y	Y	
BrCa0022	44							Y	N	Y	N	Y	
BrCa0023	65							Y	Y	Y	Y	Y	
BrCa0024	59							Y	N	Y	N	Y	
BrCa0025	58							Y	Y	Y	Y	Y	
BrCa0026	57							Y	N	Y	N	Y	
BrCa0027	65							Y	Y	Y	Y	Y	
BrCa0028	61							Y	Y	Y	Y	Y	
